# Narratives bridge the divide between distant events in episodic memory

**DOI:** 10.3758/s13421-021-01178-x

**Published:** 2021-04-26

**Authors:** Brendan I. Cohn-Sheehy, Angelique I. Delarazan, Jordan E. Crivelli-Decker, Zachariah M. Reagh, Nidhi S. Mundada, Andrew P. Yonelinas, Jeffrey M. Zacks, Charan Ranganath

**Affiliations:** 1 M.D./Ph.D. Program, University of California, Davis, Sacramento, CA USA; 2 Neuroscience Graduate Group, University of California, Davis, Davis, CA USA; 3 Center for Neuroscience, University of California, Davis, Davis, CA USA; 4 Department of Psychological and Brain Sciences, Washington University, 1 Brookings Drive, St. Louis, MO USA; 5 Department of Psychology, University of California, Davis, Davis, CA USA; 6 Memory and Aging Center, University of California, San Francisco, San Francisco, CA USA

**Keywords:** Episodic memory, Consolidation, Narratives, Recall, Event cognition

## Abstract

Many studies suggest that information about past experience, or episodic memory, is divided into discrete units called “events.” Yet we can often remember experiences that span multiple events. Events that occur in close succession might simply be linked because of their proximity to one another, but we can also build links between events that occur farther apart in time. Intuitively, some kind of organizing principle should enable temporally distant events to become bridged in memory. We tested the hypothesis that episodic memory exhibits a narrative-level organization, enabling temporally distant events to be better remembered if they form a coherent narrative. Furthermore, we tested whether post-encoding memory consolidation is necessary to integrate temporally distant events. In three experiments, participants learned and subsequently recalled events from fictional stories, in which pairs of temporally distant events involving side characters (“sideplots”) either formed one coherent narrative or two unrelated narratives. Across participants, we varied whether recall was assessed immediately after learning, or after a delay: 24 hours, 12 hours between morning and evening (“wake”), or 12 hours between evening and morning (“sleep”). Participants recalled more information about coherent than unrelated narrative events, in most delay conditions, including immediate recall and wake conditions, suggesting that post-encoding consolidation was not necessary to integrate temporally distant events into a larger narrative. Furthermore, post hoc modeling across experiments suggested that narrative coherence facilitated recall over and above any effects of sentence-level semantic similarity. This reliable memory benefit for coherent narrative events supports theoretical accounts which propose that narratives provide a high-level architecture for episodic memory.

**Supplementary Information:**

The online version contains supplementary material available at 10.3758/s13421-021-01178-x.

Most theories about memory propose that information about the past is organized according to some particular principle (Cohn-Sheehy & Ranganath, 2017). According to Tulving (1972), episodic memory, which supports the ability to recollect past events, is temporally organized. For instance, cueing people with information from a specific time can facilitate retrieving information from adjacent points in time (Estes, 1955; Howard & Kahana, 2002; Polyn et al., 2009). However, growing evidence suggests that even though people process experiences continuously, people actually draw boundaries between adjacent periods of time (Newtson, 1973), such that this continuous timeline of information is chunked into discrete units called “events” (for a review, see Zacks, 2020).

People tend to perceive event boundaries at important shifts in place, time, or situation (e.g., when a person enters or leaves a scene), and these boundaries can dissociate adjacent periods of time in memory (Radvansky & Zacks, 2011; Speer & Zacks, 2005; Zacks et al., 2007; Zacks et al., 2009; Zwaan & Radvansky, 1998). This kind of discretized, event-level organization can be adaptive for some, but not all, kinds of episodic memory retrieval. For instance, cueing people with information from a particular event facilitates retrieval of other information from that event, but it can impair the ability to retrieve information from adjacent events (DuBrow & Davachi, 2013; Ezzyat & Davachi, 2011; Horner et al., 2016; Pettijohn et al., 2016; Pettijohn & Radvansky, 2016; Swallow et al., 2011; Zacks et al., 2007). In other words, discrete events can interfere with each other during episodic memory retrieval.

If episodic memory is organized into discrete events, interference among events would pose a significant problem when one has to retrieve information that takes place over multiple events. It is often critical that we remember multiple, thematically related events that unfold over extended timescales (Brown, 2005; Kubovy, 2015). A strictly association-based account of memory would generally predict that overlapping associations across elements of different events should elicit increased interference, and therefore reduced recall performance (J. R. Anderson & Bower, 1974; J. R. Anderson & Reder, 1999; Radvansky & Zacks, 1991). If, on the other hand, information across different events can be integrated into a larger organizational unit, then such competition across events could be resolved.

It is well established that one’s ability to remember an event depends on how one comprehends the meaning of an event (Bartlett, 1932; see also Greenberg & Verfaellie, 2010; Irish & Piguet, 2013). Furthermore, there is reason to think that when people comprehend events, they construct a *narrative*: a larger unit of information that encompasses multiple events, and in which one’s comprehension of individual events is dependent on, and interrelated with, information contained within other events (Bartlett, 1932; Graesser et al., 1994; Trabasso et al., 1984; van Dijk & Kintsch, 1983; Willems et al., 2020). For instance, it is known that people track the intentions and actions of a protagonist across multiple events, and this information guides comprehension of individual events (Elman & McRae, 2019; Mandler & Johnson, 1977; Rumelhart & Ortony, 1977; Trabasso et al., 1984; van Dijk & Kintsch, 1983; Zwaan et al., 1995; Zwaan & Radvansky, 1998). Given that narratives play an important role in event comprehension, it is reasonable to suspect that narratives may shape the organization of events in memory (Bartlett, 1932).

Several theories have attempted to explain how narratives might organize relations among different events in memory (e.g., Kintsch, 1988, 1992; Radvansky, 2012; Radvansky & Zacks, 2017; Trabasso et al., 1984; Trabasso & Sperry, 1985). For example, the Event Horizon Model (EHM; Radvansky, 2012; Radvansky & Zacks, 2017) proposes that memory for information distributed across multiple events should depend on the way in which this information is associated during encoding. For instance, some events might become weakly associated because they simply share some overlapping entity (e.g., a specific character), and this overlap can cause interference when trying to retrieve specifics of any one of those events (Radvansky & Zacks, 1991). In contrast with these weaker associations, EHM proposes that people can form a network of stronger, causal associations between discrete events (i.e., when events are interrelated). EHM predicts that, because narratives provide causal links between events (see also Trabasso et al., 1984; Trabasso & Sperry, 1985), narratives are beneficial for episodic memory retrieval.

If narratives provide a high-level organization for memory, then memory should be shaped by *coherence*, the degree to which individual units of information can be interrelated within a single narrative representation to convey an overarching situation or theme (Bartlett, 1932; Graesser et al., 1994). In support of this idea, some studies have compared memory for individual narratives that are manipulated to be more or less coherent, and have found that higher coherence within a narrative is associated with better retrieval performance (Bransford & Johnson, 1972; Long et al., 2006; Thorndyke, 1977). Other studies have found that factors within narratives that contribute to coherence (e.g., semantic overlap or causal relatedness between sentences) can predict retrieval performance (Kintsch, 1988; Rumelhart, 1977; Thorndyke, 1977; Trabasso et al., 1984; Trabasso & Sperry, 1985). However, one limitation in studies of memory for narratives is that they tend to involve story events that are presented close together in time. In these instances, story events might simply be linked through their temporal proximity (Uitvlugt & Healey, 2019), rather than integrated into a narrative per se.

Intuitively, it seems that one can link events via a narrative even when they are temporally distant. For instance, in one episode of the television show *Seinfeld* (O’Keefe et al., 1997), the character Kramer first appears outside the bagel store holding a picket sign. Later in the episode, Kramer announces that he is leaving a dinner party to go bake bagels. Although viewers see these scenes scattered at different points during the episode, they can infer that Kramer was striking, but eventually broke the strike and returned to work at the bagel store. In other words, one can recall these temporally separated, but interrelated, events in terms of a coherent narrative.

If EHM is correct (Radvansky, 2012; Radvansky & Zacks, 2017), even temporally separated events should benefit from incorporation into a coherent narrative. That is, pairs of temporally separated events that form a coherent narrative should be recalled in greater detail than pairs of temporally separated events that do not form a coherent narrative, even if both types of event pairs have some shared features like a specific character. EHM suggests that people can form coherent links between temporally separated events during memory encoding, and this should support subsequent memory retrieval.

An alternative possibility is that some post-encoding memory consolidation process is required to integrate temporally distant events over a delay, either through some time-dependent (Moscovitch et al., 2016; Winocur & Moscovitch, 2011) or sleep-dependent memory consolidation process (Frankland & Bontempi, 2005; Lewis & Durrant, 2011). In support of this idea, a recent study by Liu and Ranganath (2019) found that temporally distant, semantically related pictures of objects can initially exhibit retrieval interference, whereas after sleep, these objects paradoxically exhibit retrieval facilitation. Liu and Ranganath concluded that sleep-dependent consolidation integrates semantically related information that is temporally distant (Lewis & Durrant, 2011); however, they did not investigate memory for narratives. It is not clear whether this kind of consolidation for temporally distant, semantically related information would extend to narrative events.

The goal of the present study was to determine the extent to which recall of separated events can benefit from incorporation into a coherent narrative, and to identify whether this process might require sleep-dependent memory consolidation. We conducted three behavioral experiments to test the idea that narratives can facilitate event recall, even when the events that form the narrative occur in distinct temporal contexts (i.e., are temporally distant). We created a set of fictional stories (see Supplemental Dataset 1) that included events with characters that overlapped across stories. Events involving some recurring characters could be integrated into a broader, coherent narrative, whereas other recurring characters were involved in separate, unrelated events (see Fig. 1). Later, participants were asked to recall events involving each recurring character, and we contrasted memory for story events that included characters who were involved in coherent versus unrelated narratives.

**Fig. 1:**
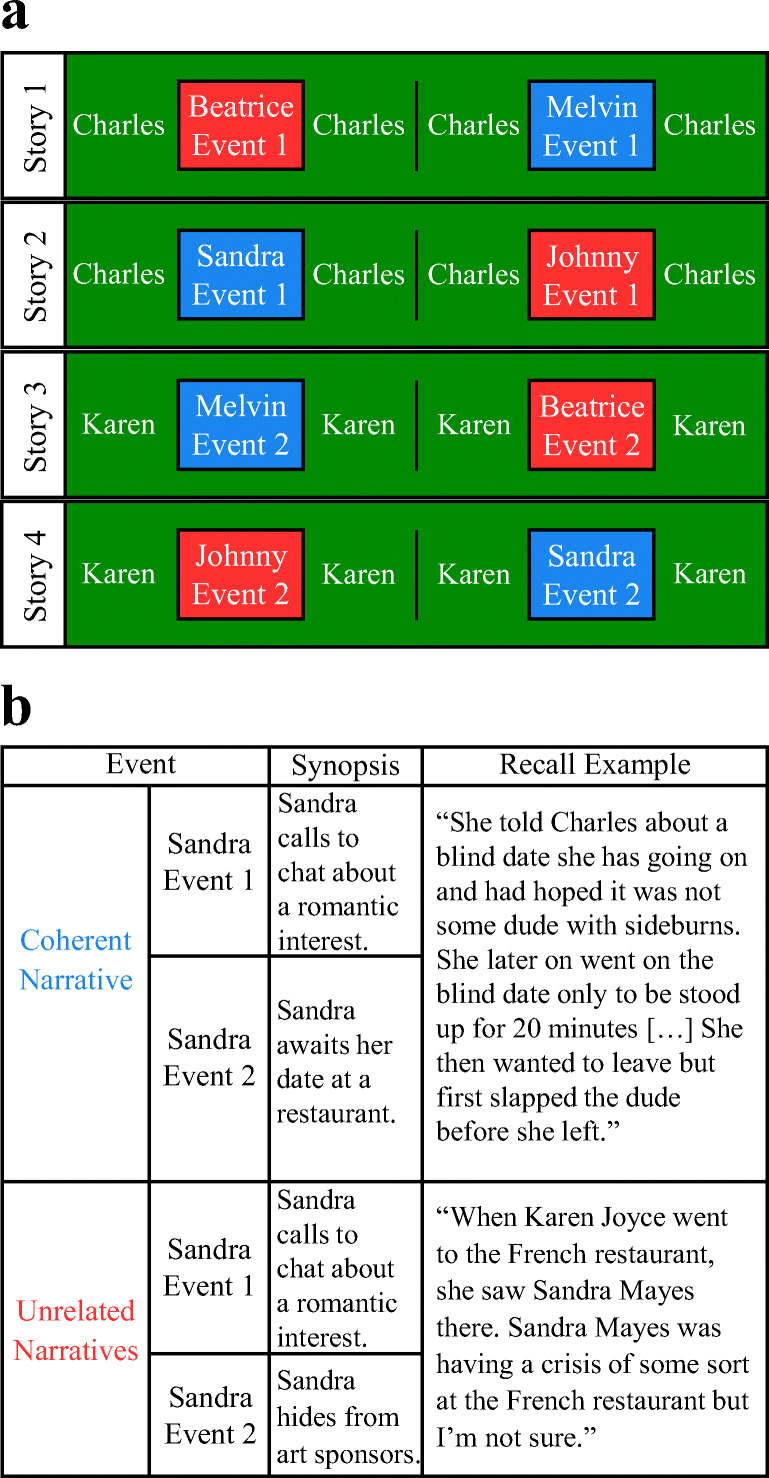
Sideplot coherence paradigm. **a** Stimulus design and presentation: Four fictional stories were presented serially as audio clips (240s each), in the context of long stories about one of two main protagonists (Charles or Karen; “main plot events” in Green). Events involving key side-characters (Beatrice, Melvin, Sandra, Johnny; 40s each) did not relate to the main plot events (they were “sideplots”). However, for two side-characters (Blue boxes), two temporally-distant events could form one Coherent Narrative (e.g. Sandra Events 1 and 2). In contrast, for two other side-characters (Red boxes), Events 1 and 2 belonged to Unrelated Narratives (e.g. Johnny). **b** Examples of narrative events: Synopses and recall examples (of varying success) are provided for two possible pairs of events for Sandra, one which is Coherent, and one which is Unrelated. For each participant, side-characters were randomly assigned to either the Coherent Narrative or Unrelated Narratives conditions

We hypothesized that temporally distant events that form a coherent narrative would be integrated in memory, and would therefore be recalled in greater detail than unrelated events that could not be integrated into a larger narrative. Furthermore, we investigated whether consolidation is necessary to integrate temporally distant events into a coherent narrative. We predicted that if consolidation is necessary for integrating temporally distant events, coherence effects would only be observed after a 24-hour delay (Experiments 1 and 3) or overnight sleep (Experiment 2). In contrast, we predicted that if post-encoding consolidation processes are not necessary for integration, coherence effects would also be observed during immediate recall. Alternatively, consolidation could augment coherence effects, augment retrieval performance regardless of coherence, or have no effect whatsoever.

## Experiment 1

We first investigated whether the opportunity to construct a coherent narrative across distant events would benefit recall, and whether this benefit would depend on post-encoding processes. We presented subjects with custom fictional stories and assessed their recall either immediately (Immediate Recall, *N* = 36) or after a 24-hour delay (Delayed Recall, *N* = 36). As described above, in accordance with the EHM and related theories, we hypothesized that temporally separated events that form a coherent narrative would be recalled in greater detail than temporally separated events that do not form one unified narrative. Furthermore, we hypothesized that if post-encoding processes (i.e., consolidation) are necessary to integrate temporally separated events into a larger narrative, then only the Delayed Recall group would exhibit any recall benefit for distant events that form a coherent narrative.

### Methods

#### Participants

Seventy-eight participants, 18–30 years old (*M* = 20.2 years, *SD* = 2.1 years, 47 female) were initially recruited from a pool of undergraduate students enrolled in psychology courses at the University of California, Davis, who earned course credit for their participation. Using the UC Davis Psychology Research Participation System, participants could opt to sign up for either one 90-minute session (Immediate Recall) or two 30-to-60-minute sessions that took place 24 hours apart (Delayed Recall). Both groups earned equivalent course credits, and neither group was informed of the retention interval manipulation. Because our task centered on audio recordings of fictional narratives in the English language, we used responses from a prescreening survey administered by the University of California, Davis Psychology Department to include only persons who (a) had normal hearing, (b) used English as their primary language, (c) had experience with English before age 5, and (d) were born in the United States or lived in the United States for at least 15 years prior to recruitment. We excluded subjects for the following reasons: (a) they were absent from the Recall phase (Day 2) of the Delayed Recall condition (*N* = 3), or (b) they did not follow instructions on the Recall task (*N* = 3).

We aimed to recruit samples of *N* = 36 participants for analysis of each Retention Interval group (*N* = 72 total); therefore, we continued to recruit participants until we had full samples without additional exclusion. This sample size was selected based on a power analysis of a within-subjects pilot study (Delayed Recall only) that revealed preferentially higher recall of distant events that could form a coherent narrative (Cohen’s *d* = 0.48, power = 80%, alpha = 0.05).

#### Stimulus design

The stimuli and overall design are depicted in Fig. 1. We constructed four fictional stories in which we manipulated whether temporally distant events could form a coherent narrative (see Fig. 1a; Supplemental Dataset 1). Story audio was scripted, recorded, and thoroughly edited by the first lead author (B.I.C.S.) to ensure equivalent lengths for all sentences (5 s each), events (eight sentences/40 s each), and stories (six events/240 s each), and to avoid any differences in perceptual distinctiveness (see Supplemental Methods). Two of these stories are centered on a character named Charles, who is attempting to get a big scoop for a newspaper, and two are centered on a character named Karen, who is attempting to find employment as a chef.

To examine the effects of narrative-level organization on memory, we incorporated events involving key “side-characters” into each story. Two stories involved “Beatrice” and “Melvin” as side-characters, and two involved “Sandra” and “Johnny.” Each side-character appeared in two distinct events that occurred 6–12 minutes apart (i.e., they were temporally distant), in disparate contexts across two unrelated stories. For instance, Sandra’s first event occurs in Story 2, when she calls Charles, who is sitting at a park at noon on a Tuesday. Sandra’s second event occurs in Story 4, when Karen runs into her at a French restaurant, at early evening the next day. Events involving the side-characters were tangential to adjacent “main plot events” which centered on Charles and Karen (i.e., they were “sideplots”).

Critically, for two side-characters, these sideplot event pairs could form a Coherent Narrative (CN) about one particular situation involving that side-character. In contrast, for the other two side-characters, each sideplot event described a different situation involving that side-character, such that the two sideplot events could not easily form a singular coherent narrative (Unrelated Narratives [UN]). Because the stimulus design controlled for several other features that could support integration of sideplot events (temporal proximity, contextual similarity, attention to intervening main plot events), only CN events, and not UN events, could be easily integrated into a larger narrative.

We sought to control for any effects of specific event content or character identity that could confound the coherence manipulation, by randomizing sideplot event content across subjects. For each side-character, we created two alternate pairs of CN events (e.g., Sandra Events 1 and 2, Version A; Sandra Events 1 and 2, Version B) that had similar syntax. For a given subject, two side-characters were randomly selected to be CN, and two side-characters were randomly selected to be UN. If a side-character was selected to be CN, one of the two possible CN event pairs was selected (e.g., Sandra Events 1 and 2, Version A; see Fig. 1b). If a side-character was selected to be UN, the two events were drawn from different possible CN event pairs, such that they belonged to unrelated narratives (e.g., Sandra Event 1, Version A, and Event 2, Version B; see Fig. 1b). However, each story always contained one CN and one UN event (e.g., in Fig. 1a, Sandra is CN and Johnny is UN). This approach resulted in 32 possible arrangements of sideplot events.

#### Behavioral tasks

We presented the four fictional stories once through in chronological order (1–4), and then tested free recall of particular characters, with or without a 24-hour retention interval between story presentation and recall (i.e., 1 vs. 2 visits to the lab). Prior to presenting stories, we also included brief tasks that familiarized participants with character names, with the aid of memorable faces (Bainbridge et al., 2013), such that we could use character names as recall cues. The experiment order was as follows: (1) familiarization tasks (see Supplemental Methods); (2) story presentation; (3) retention interval; (4) recall task. Familiarization and story presentation tasks were implemented in MATLAB Version R2015a (https://www.mathworks.com/products/matlab.html) using Psychophysics Toolbox 3 (https://www.psychtoolbox.org), and recall was assessed using Qualtrics (https://www.qualtrics.com/).

***Story presentation****.* Participants were instructed verbally and onscreen that they would hear fictional clips involving characters from the familiarization tasks, and that they should devote their full attention and imagination to the clips as if they were listening to a book they enjoyed. Furthermore, they would later be asked to remember these stories in detail. For each participant, one of the 32 sideplot arrangements was randomly selected, and stories were presented binaurally through over-ear headphones, with only a white fixation cross present onscreen. After a story elapsed for four minutes, onscreen text indicated that participants should press the space bar to start the next story. Participants could pause for a few seconds if necessary during this screen. After all stories were presented, onscreen text indicated that the task was complete.

***Retention interval****.* Immediate Recall participants completed this study in a 1-day protocol in which recall immediately followed story presentation, with no intervening task. Delayed Recall participants completed this study in a 2-day protocol in which participants left after story presentation on Day 1, and returned 24 hours later to complete recall on Day 2.

***Recall task****.* Each character cue was presented on a separate survey screen with a box for typing recall. We cued CN and UN side-characters in a randomized order, and then the two main protagonists in a randomized order. For side-character cues, we included their relations to main protagonists, to encourage recall of both sideplot events (e.g., “Melvin Doyle [Charles Bort’s neighbor, Karen Joyce’s friend]”). Participants were instructed to type everything they could remember involving the particular character, from all stories, in as much detail as possible, and for at least 5 minutes, and they were encouraged to continue typing if they remembered additional details. To encourage a continuous, extensive recall process which would be akin to verbal recall, we additionally instructed participants to type their thoughts “as they immediately come to mind, without planning, editing, or revising,” except if they needed to immediately fix a typo. We did not instruct participants to recall events in any particular order, nor did we instruct participants to integrate information between events. Although we did not initially predict any differences in recall between specific events (Event 1 vs. Event 2), these instructions were intended to prevent biasing subjects to recall one event versus another.

#### Recall scoring

To quantify recall performance, we adapted a well-characterized scoring method from the Autobiographical Memory Interview (Diamond et al., 2020; Levine et al., 2002). Recall data were scored in a blinded fashion by segmenting each participant’s typed recall into meaningful detail units (Levine et al., 2002), and then determining how many of these details could be verified within specific story events (Supplemental Datasets 2–3). Briefly, each recall transcript was segmented into the smallest meaningful unit possible (“details”), and details were assigned labels that describe their content. We primarily sought to label details that could be verified within the story text (“Verifiable Details”). Verifiable Details are details that specifically refer to events that center on the cued character (i.e., neither inferences about cued events, nor “external details” about other characters or events), which are recalled with some degree of certainty (e.g., not preceded by “I think” or “Maybe”), and which do not merely restate recall cues or other previously recalled details for a given cue.

We were also interested to discern recall performance for each sideplot event (Events 1 or 2). For each CN or UN cue, Verifiable Details that referred to one particular event were labeled as “Event 1” or “Event 2.” If a Verifiable Detail could have originated from either Event 1 or Event 2, it was scored as “Either”; these details were rare (X̄ = 0.07 details per cue, *SD* = 0.32 details/cue, max = 3.5 details/cue). Finally, if a Verifiable Detail merged information from both Event 1 and Event 2, it was marked as “Integrated”; these details were both rare and only observed for CN events (X̄ = 0.07 details/cue, *SD* = 0.39 details/cue, max = 4.5 details/cue). To ensure that analyzed details are event-specific, “Either” and “Integrated” details have been excluded from all reported analyses.

Three raters (AGl, NM, SH) who were blinded to the experimental hypotheses and coherence conditions were trained on the scoring criteria described above. Raters practiced scoring recall transcripts from pilot experiments, and then they scored recall transcripts from this study as it was collected. Two to three raters scored each participant’s recall. Raters had the opportunity to discuss their scoring approach with each other and to consult the lead authors (B.I.C.S., A.I.D.) regarding any difficulties with the scoring method. Once all scoring was complete, interrater reliability (IRR) for CN and UN events was high (mean Pearson’s *r =* .83), taking into account how many Verifiable Details were scored per event label (Event 1, Event 2, Either, Integrated), per character, and per participant. For comparison purposes, raters also scored recall of events which centered on main protagonists (Charles, Karen), but did not involve the cued side-characters (i.e., “main plot events”). IRR for total verifiable details recalled from main plot events for each main protagonist cue was high (mean Pearson’s *r* = .82). All reported analyses are based on counts of Verifiable Details averaged across raters.

#### Data analysis

Statistical analysis was performed in R (https://www.r-project.org/), using the Afex package (https://github.com/singmann/afex) for analyses of variance (ANOVAs). For all ANOVAs, we performed planned two-tailed *t* tests for pairwise contrasts corresponding to significant *F* tests. Where necessary, ANOVAs implemented Greenhouse–Geisser corrections for nonsphericity. For additional interpretation of findings, we computed Bayes factors (see Wagenmakers et al., 2016) using Bayes factor *t* tests (ttestBF function within BayesFactor in R; r-scale set to default, √2/2; https://www.rdocumentation.org/packages/BayesFactor; see also Rouder et al., 2009), to determine the level of evidence for or against the null hypothesis (i.e., effect size equal to zero). To follow up on main effects, Bayes factor *t* tests compared recall between two conditions of interest (e.g. CN vs. UN events). To follow up on interactions, Bayes factor *t* tests compared recall differences (i.e., recall of CN-minus-UN events) across groups (i.e., Immediate Recall vs. Delayed Recall). Bayes factor magnitudes were interpreted to indicate the relative strength of evidence (Kass & Raftery, 1995) in favor of our primary hypothesis (BF_10_) or in favor of the null hypothesis (BF_01_ = 1/BF_10_).

### Results and discussion

Our primary aim was to determine whether coherent narratives provide a retrieval benefit for temporally distant events, and whether this benefit manifests over a delay. In order to investigate these ideas, we primarily analyzed recall for pairs of sideplot events, because these pairs were specifically manipulated to either form a coherent narrative (CN) or not (UN). Main plot events were not designed to test these hypotheses, and instead served to provide an additional assessment of recall ability for each experimental group.

**Fig. 2:**
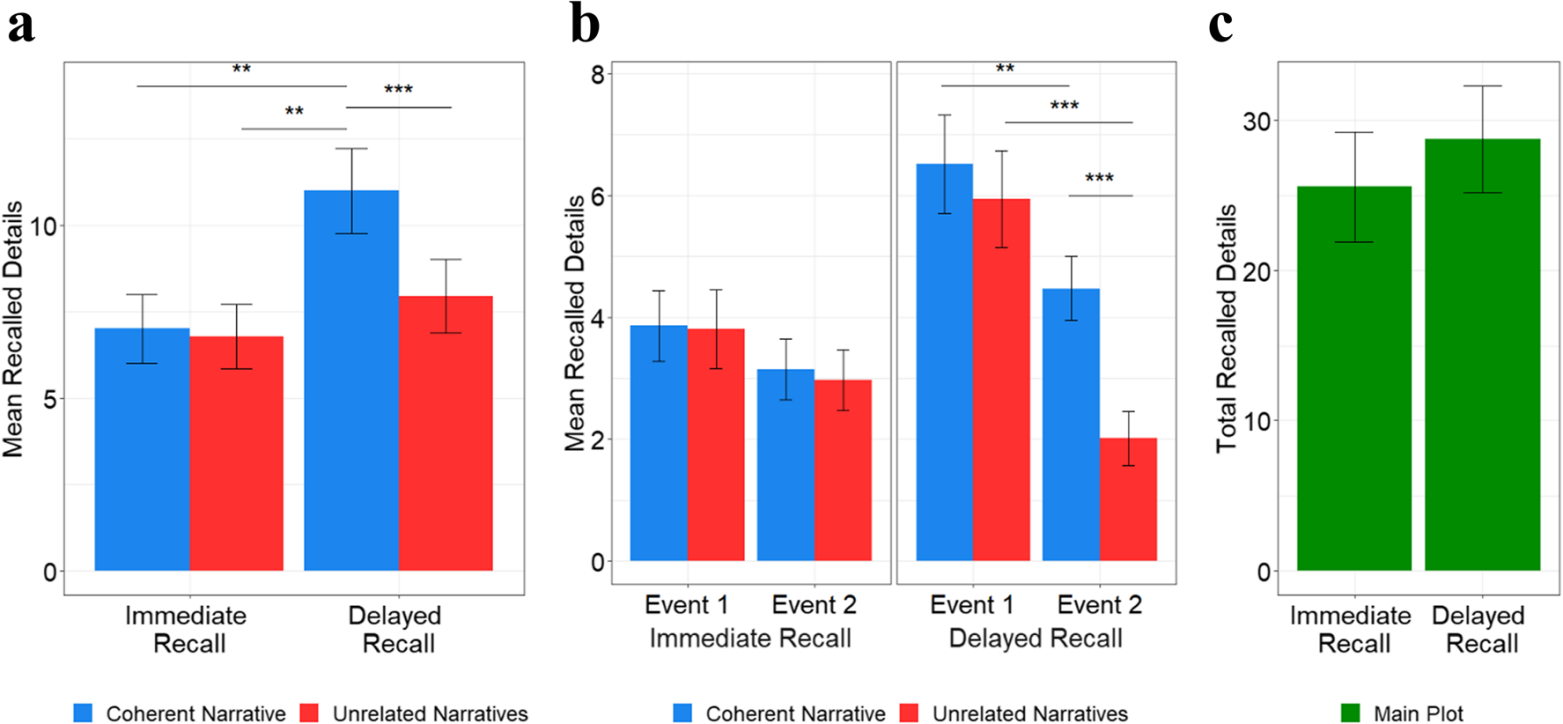
Experiment 1 Results: Delayed recall benefit for Coherent Narrative events. **a** Overall recall of sideplot events: verifiable recalled details are summed for each side-character, and binned and averaged by Coherence (Coherent Narrative, Unrelated Narratives) and Retention Interval (Immediate Recall, Delayed Recall). **b** Recall of individual sideplot events: similar to **a**, except recalled details are binned and averaged by Event Number (1, 2) and Coherence, within each Retention Interval group. **c** Recall of main plots: verifiable recalled details for main plot events are summed for each main protagonist, and then binned and averaged by Retention Interval group. Key: Bars = mean recalled details (+/- standard error of the mean), brackets = significant t-tests: ** = *p* < 0.01, *** = *p* < 0.001

Our primary analysis focused on sideplot event recall at each delay (see Fig. 2). We hypothesized that distant events that formed one coherent narrative should be more highly recalled than events that did not (CN > UN). Furthermore, if such a benefit is dependent on post-encoding consolidation, it would only be observed after a delay (CN > UN at Delayed Recall, not Immediate Recall). We performed a 2 × 2 ANOVA, incorporating a within-subjects factor of Coherence [CN vs. UN] and a between-subjects factor of Retention Interval [Immediate Recall vs. Delayed Recall]. This comparison revealed a significant effect of Coherence, *F*(1, 70) = 9.00, η_G_^2^ = .02, *p* = .004, and a non-significant effect of Retention Interval, *F*(1,70) = 3.39, η_G_^2^ = .04, *p* = .07. The Bayes factor for the main effect of Coherence (BF_10_ = 5.83) suggested that there was substantial evidence in favor of our first hypothesis: that Coherent Narrative events would be recalled in greater detail than Unrelated Narrative events.

These main effects were qualified by a significant Retention Interval × Coherence interaction, *F*(1, 70) = 6.65, η_G_^2^ = .01, *p* = .01. Pairwise contrasts revealed that more details were recalled for CN than UN events within the Delayed Recall group, *t*(70) = 3.95, *p* = .0002, Cohen’s *d* = .66. Interestingly, CN events within the Delayed Recall group were recalled better than any sideplots within the Immediate Recall group: CN/Delayed Recall > CN/Immediate Recall, *t*(90.7) = 2.65, *p* = .0095, Cohen’s *d* = 0.62; CN/Delayed Recall > UN/Immediate Recall, *t*(90.7) = 2.80, p = .006, Cohen’s *d* = 0.66. No other pairwise contrasts were significant (all *t*s < 1.0). In other words, CN events were not only more highly recalled than UN events at a 24-hour delay; they were also more highly recalled than either CN or UN events at immediate recall. The Bayes factor for the interaction (BF_10_ = 3.95) suggested that there was substantial evidence in favor of our second hypothesis: that a benefit of Coherence on recall would be modulated by a delay (i.e., by consolidation). However, as we will describe later, this interaction was not replicated in a subsequent experiment.

To follow up on this result, we investigated the extent to which narrative coherence affected recall separately for the first and second sideplot events (see Fig. 2b). Within each group, we modeled sideplot event recall using a 2 × 2 ANOVA with within-subjects factors of Coherence [CN vs. UN] and Event Number [1 vs. 2]. For the Delayed Recall group, results showed that overall recall was higher for CN than UN events (significant main effect of Coherence), *F*(1, 35) = 13.60, η_G_^2^ = 0.04, *p* = 0.0008, and higher for Event 1 than for Event 2 (significant main effect of Event), *F*(1, 35) = 40.37, η_G_^2^ = 0.13, *p* < .0001. These main effects were qualified by a significant Coherence × Event Number interaction, *F*(1, 35) = 4.17, η_G_^2^ = 0.01, *p* = .05. This interaction reflected that recall of CN and UN events did not significantly differ at Event 1, *t*(69) = 0.921, *p* = .36, Cohen’s *d* = 0.15, whereas there was a significant recall difference between CN and UN Events at Event 2, *t*(69) = 3.97, *p* = .0002, Cohen’s *d* = 0.66. For the Immediate Recall group, there were no significant effects of Coherence, *F*(1, 35) = 0.10, η_G_^2^ = 0.0003, *p* = .75, nor Event, *F*(1, 35) = 2.88, η_G_^2^ = 0.01, *p* = .10, nor a significant Coherence × Event Number interaction, *F*(1, 35) = 0.03, η_G_^2^ = <0.0001, *p* = 0.86. As such, only the Delayed Recall group exhibited any impact of narrative coherence on specific event recall.

Importantly, Retention Interval was a between-groups condition. In other words, it was possible that any differences in recall based on Retention Interval (including the Retention Interval × Coherence interaction) could have been driven by differences in recall ability. For instance, one might presume that forgetting occurs even over a 24-hour delay, and therefore the Immediate Recall group would more likely recall more details than the Delayed Recall group. To address this possibility, we tested whether main plot events were more highly recalled by either group. There was no statistical evidence for this, *t*(70) = −0.775, *p* = 0.44, Cohen’s *d* = 0.15, and in fact, the Delayed Recall group exhibited numerically (but nonsignificantly) higher recall than the Immediate Recall group (see Fig. 2c). However, the Bayes factor for this difference (BF_10_ = 0.29, BF_01_ = 3.43) suggested that, instead, there was substantial evidence in favor of the null hypothesis (i.e., zero effect of Retention Interval on main plot recall). Because this trend was unexpected, we investigated whether it was driven by outliers in either group. There were no clear main plot recall outliers within the Immediate Recall group; however, within the Delayed Recall group, four subjects exhibited higher recall than 1.5 times the interquartile range (75^th^-minus-25^th^ percentile). Removing these outlier subjects resulted in a lower average for main plot recall in the Delayed Recall group (mean = 23.4 details recall), resulting in a trend towards greater recall in the Immediate Recall group; however, this trend was not significant, *t*(60) = 0.52, *p* = .60, Cohen’s *d* = 0.12, BF_01_ = 3.60. Furthermore, removing these outlier subjects did not change the overall pattern of findings for sideplot recall (see Supplemental Results).

In summary, results of Experiment 1 were consistent with the idea that narrative coherence facilitates recall of temporally distant events, but only after a 24-hour delay; this provided preliminary support for the consolidation hypothesis. However, these results were also somewhat counterintuitive, as we did not see any clear indications that overall recall performance was worse for the Delayed Recall group. Therefore, we sought to follow up on these findings by matching retention interval lengths and assessing the specific role of sleep, which would provide a potentially more specific test for the role of consolidation in forming coherent narratives across events. To plan the subsequent experiment, we conducted a power analysis, which revealed that *N* = 45 participants per group would be required to replicate the Retention Interval × Coherence interaction (Cohen’s *d* = 0.6, power = .80, α = .05).

## Experiment 2

Experiment 2 expanded upon the findings of Experiment 1 to investigate the role of sleep in memory for coherent narrative events. This experiment largely replicated the methods of Experiment 1. However, the Retention Interval manipulation was based on the presence or absence of sleep over a 12-hour delay (Sleep vs. Wake groups, *N* = 45 each), matching retention interval length. If our effects in Experiment 1 were driven by sleep-dependent memory consolidation, then we would expect a Coherence × Retention Interval interaction to replicate in the context of a sleep study. Specifically, we hypothesized that if sleep-dependent consolidation is necessary to integrate temporally separated events into a larger narrative, then only the Sleep group would exhibit any recall benefit for distant events that form a coherent narrative. Alternatively, in accordance with EHM and related theories, it was possible that encoding and/or retrieval processes might be sufficient to integrate temporally separated events into a larger narrative (i.e., without sleep-dependent consolidation). If so, distant events that form a coherent narrative would be recalled in greater detail than distant events which could not form one unified narrative, in both the Sleep and Wake groups.

### Methods

#### Participants

One-hundred-fifty-three participants, 18–37 years old (*M* = 20.1 years, *SD* = 2.3 years, 102 female, 55 male) were initially recruited from undergraduate psychology courses at the University of California, Davis, and were compensated course credit for their participation. Using the UC Davis Psychology Research Participation System, participants could opt to sign up for two experimental sessions which were separated by 12 hours overnight (Sleep) or 12 hours during the daytime (Wake). Both groups earned equivalent course credits, and neither group was informed of the sleep-versus-wake manipulation. Inclusion criteria were identical to those of Experiment 1. We excluded participants for the following reasons: (a) they were absent from the Recall phase (*N* = 17); (b) they did not follow instructions on the Recall task (*N* = 8); (c) they did not follow instructions during the Encoding task (*N* = 5); (d) they napped during the intervening delay period of the Wake condition (*N* = 9); (e) they reported less than 5 hours of sleep on average for the 3 nights preceding the Encoding phase (Session 1), or they slept less than 5 hours either the night before the Encoding phase (Session 1) or the Recall phase of the Sleep condition (*N* = 19); or (f) technical difficulties (*N* = 5). The Experiment 1 power analysis indicated that *N* = 45 subjects per group were needed to replicate a Retention Interval × Coherence interaction (*N* = 90 total), therefore we continued to recruit participants until we had full samples without additional exclusion.

#### Stimulus design, behavioral tasks, and data analysis

These were near-identical to Experiment 1, except for retention interval conditions. Familiarization and story presentation tasks were run in a newer MATLAB version, R2019a. Participants also completed standardized sleep questionnaires, which revealed comparable scores between groups (see Supplemental Methods, Table S1).

***Retention interval****.* The study was completed in two sessions, separated by 12-hour intervals of wake (daytime) or sleep (overnight). Wake participants completed story presentation at morning (7 a.m. or 8 a.m.), and recall at night (7 p.m. or 8 p.m.). Sleep participants completed story presentation at night (7 p.m. or 8 p.m.), and recall the following morning (7 a.m. or 8 a.m.).

#### Recall scoring

**Fig. 3:**
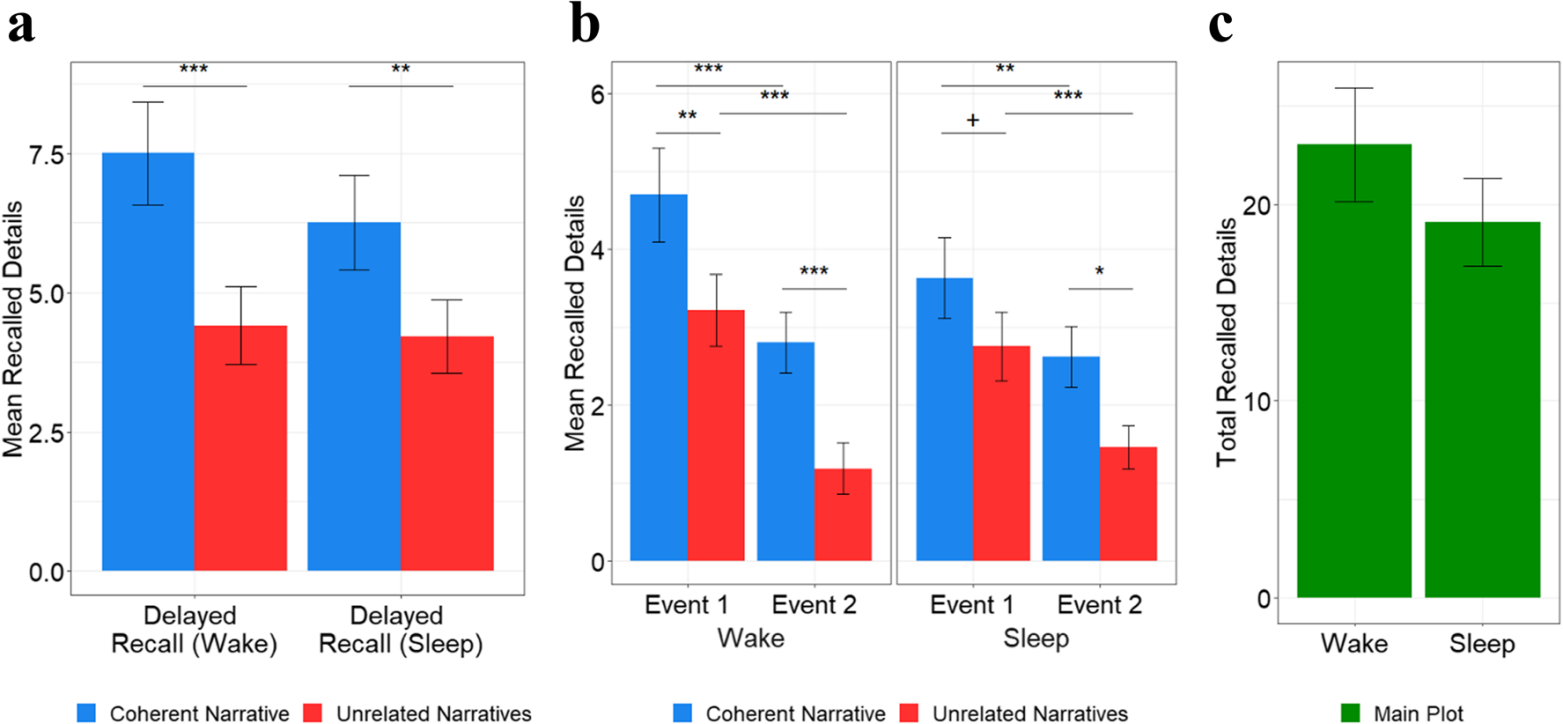
Experiment 2 Results: Recall benefit for Coherent Narrative events in both Wake and Sleep conditions. **a** Overall recall of sideplot events: verifiable recalled details are summed for each side-character, and binned and averaged by Coherence (Coherent Narrative, Unrelated Narratives) and 12-hour Retention Interval group (Delayed Recall-Wake, Delayed Recall-Sleep). **b** Recall of individual sideplot events: similar to **a**, except recalled details are binned and averaged by Event Number (1, 2) and Coherence, within each Retention Interval group. **c** Recall of main plots: verifiable recalled details for main plot events are summed for each main protagonist, and then binned and averaged by Retention Interval group. Key: Bars = mean recalled details (+/- standard error of the mean), brackets = significant t-tests: += *p* <0.10, * = *p* <0.05, ** = *p* <0.01, *** = *p* <0.001

A new group of raters (AGa, EM, JD, MD) was trained to score recall using the same scoring procedure as Experiment 1. Final IRR was high for sideplot events (mean Pearson’s *r =* .88) and for main plot event totals (mean Pearson’s *r* = .96). “Either” and “Integrated” details were counted, but not included in further analysis (i.e., not included in Fig. 3). “Either” details were rare (X̄ = 0.08 details/cue, *SD* = 0.35 details/cue, max = 3 details/cue), and “Integrated” details were both rare and only observed for Coherent sideplots (X̄ = 0.11 details/cue, *SD* = 0.49 details/cue, max = 4 details/cue).

### Results and discussion

As in Experiment 1, our primary analyses focused on sideplot events that differed based on whether they formed coherent narratives (CN vs. UN). If the delay-dependent benefit of narrative coherence on memory was driven by sleep-dependent consolidation, then we would expect an interaction, such that recall would be higher for CN than for UN events in the Sleep group, but not in the Wake group. To test these predictions, our primary analysis focused on whether recall of CN and UN events differed between Wake and Sleep groups (see Fig. 3a). We modeled sideplot event recall using a 2 × 2 ANOVA, with a within-subjects factor of Coherence [CN vs. UN] and a between-subjects factor of Retention Interval [Wake vs. Sleep]. This comparison revealed a significant main effect of Coherence, *F*(1, 88) = 23.59, η_G_^2^ = 0.06, *p* < .0001. The Bayes factor for the main effect of Coherence (BF_10_ = 1253.35) suggested that there was decisive evidence in favor of our first hypothesis, that Coherent Narratives would benefit recall of temporally distant events.

However, neither an effect of Retention Interval, *F*(1, 88) = 0.54, η_G_^2^ = 0.005, *p* = .47, nor a Retention Interval × Coherence interaction, *F*(1, 88) = 0.98, η_G_^2^ = 0.002, *p* = .32, were significant. Pairwise contrasts to break down the main effect of Coherence revealed that more details were recalled for CN than UN events, in both the Wake group, *t*(88) = 4.14, *p* = .0001, Cohen’s *d* = 0.62, and the Sleep group, *t*(88) = 2.73, *p* = .008, Cohen’s *d* = 0.41. In other words, both types of 12-hour retention interval groups demonstrated a recall benefit for coherent narrative events, but this recall benefit was not driven by sleep. This suggests that sleep-dependent consolidation was not necessary to form a coherent narrative across events. However, the Bayes factor for the interaction (BF_10_ = 0.39, BF_01_ = 2.58) did not provide substantial evidence in favor of the null hypothesis (i.e., that sleep has *zero* impact on recall of Coherent Narrative events).

To follow up on this result, we investigated the extent to which narrative coherence affected recall separately for the first and second sideplot events (see Fig. 3b). In line with our modeling approach for Experiment 1, within each group, we modeled sideplot event recall using a 2 × 2 ANOVA with within-subjects factors of Coherence [CN vs. UN] and Event Number [1 vs. 2]. Results showed that overall recall was higher for CN than UN events (significant main effect of Coherence) in both the Wake group, *F*(1, 44) = 15.77, η_G_^2^ = 0.06, *p* = .0003, and the Sleep group, *F*(1, 44) = 6.36, η_G_^2^ = 0.03, *p* = .02, and higher for Event 1 than for Event 2 (significant main effect of Event Number) in both the Wake group, *F*(1, 44) = 37.42, η_G_^2^ = 0.09, *p* < .0001, and the Sleep group, *F*(1, 44) = 26.38, η_G_^2^ = 0.04, *p* < .0001. Unlike Experiment 1, no significant Coherence × Event Number interaction was observed in either the Wake group, *F*(1, 44) = 0.08, η_G_^2^ = 0.0001, *p* = .79, or the Sleep group, *F*(1, 44) = 0.57, η_G_^2^ = 0.001, *p* = .45. Because interactions were not statistically significant, limited inference could be drawn about whether the Coherence benefit was specific to Event 2, or extended to both Events 1 and 2.

Finally, we used main plot recall to determine whether there were any overall differences in recall ability between groups. Although the primary aim of this analysis was to assess any cohort effects on recall (in comparison with Experiment 1), there are also theoretical reasons to suspect that sleep might either benefit subsequent recall, or result in forgetting (Poe, 2017; Sara, 2017). We compared main plot recall between Wake and Sleep groups (see Fig. 3c). This comparison revealed that there was numerically higher main plot recall in the Wake group than the Sleep group, however, this difference was not significant, *t*(88) = −1.17, *p* = 0.24, Cohen’s *d* = 0.25, BF_10_ = 0.40, BF_01_ = 2.50. In other words, there was no clear sleep-dependent difference in overall recall ability.

The findings of Experiment 2 suggest that sleep-dependent consolidation does not explain the recall benefit for coherent narrative events. Moreover, this study was designed to replicate and extend the Retention Interval × Coherence interaction from Experiment 1. Because there was no such interaction in this experiment, it was necessary to conduct a direct replication of Experiment 1, to provide a final adjudication between EHM and consolidation hypotheses.

## Experiment 3

The findings of Experiment 1 provided initial support for the hypothesis that post-encoding memory consolidation is necessary to facilitate memory for temporally separated events that form a coherent narrative. In contrast, the findings of Experiment 2 suggested that sleep-dependent consolidation was not necessary to facilitate memory for coherent narrative events. Because the findings of Experiments 1 and 2 found conflicting evidence for and against the consolidation hypothesis, it was important to determine whether the findings of Experiment 1 would replicate in a separate group of participants. Therefore, Experiment 3 implemented identical procedures and a power analysis-determined sample size to replicate the Retention Interval × Coherence interaction from Experiment 1.

### Methods

#### Participants

Ninety-seven participants, 18–30 years old (*M* = 20.9 years, *SD* = 1.5 years, 78 female) were initially recruited from undergraduate psychology courses at the University of California, Davis, and earned course credit for their participation. Using the UC Davis Psychology Research Participation System, participants could opt to sign up for either one 90-minute session (Immediate Recall) or two 30-to-60-minute sessions that took place 24 hours apart (Delayed Recall). Both groups earned equivalent course credits, and neither group was informed of the retention interval manipulation. Inclusion criteria were identical to Experiment 1. We excluded participants for the following reasons: (a) they did not follow instructions on the Recall task (*N* = 5); (b) technical difficulties (*N* = 2). The Experiment 1 power analysis indicated that *N* = 45 subjects per group were needed to replicate a Retention Interval × Coherence interaction (*N* = 90); therefore, we continued to recruit participants until we had full samples without additional exclusion.

#### Stimulus design and behavioral tasks

These were generally identical to Experiment 1. Familiarization and story presentation tasks were run in MATLAB version R2019a (https://www.mathworks.com/products/matlab.html).

#### Recall scoring

**Fig. 4:**
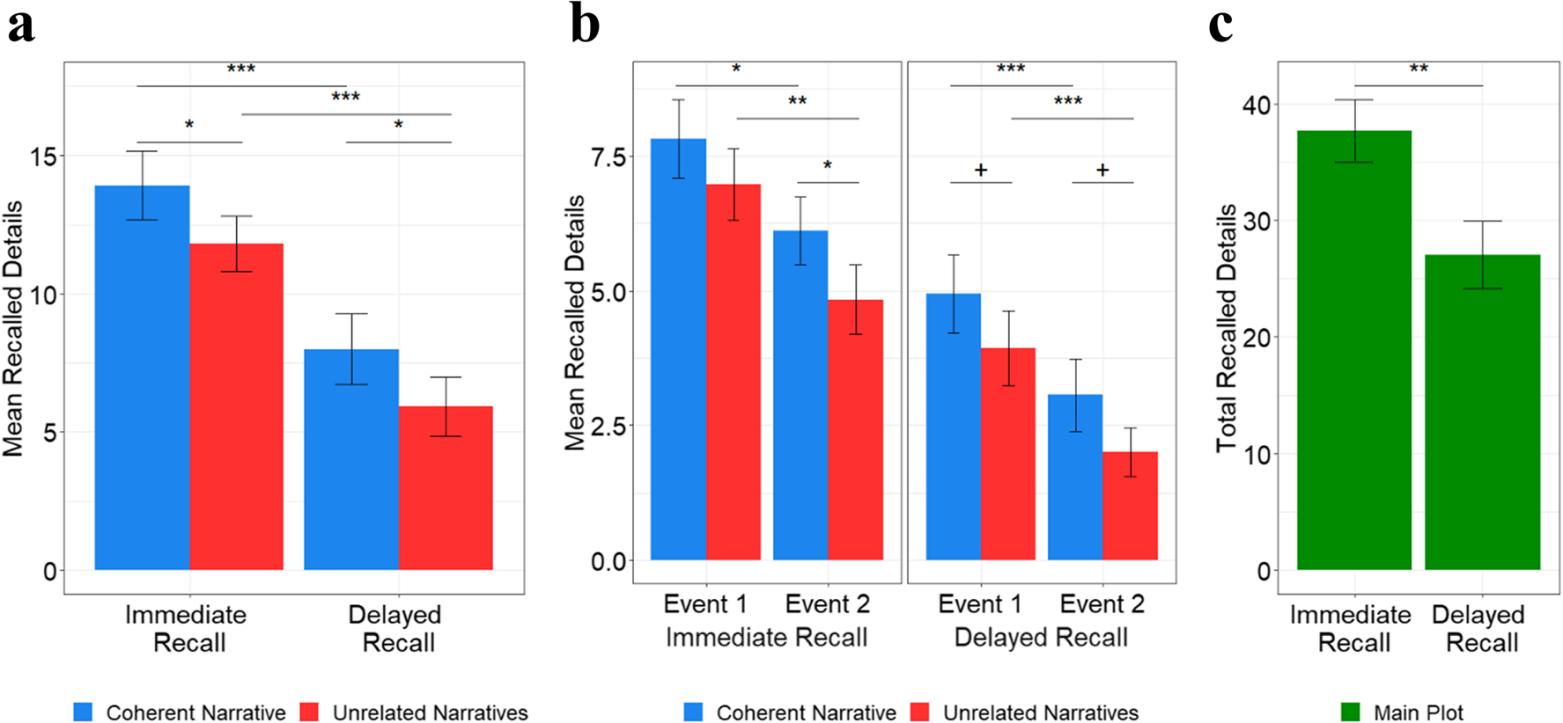
Experiment 3 Results: Recall benefit for Coherent Narrative events in both Immediate and Delayed conditions. **a** Overall recall of sideplot events: verifiable recalled details are summed for each side-character, and binned and averaged by Coherence (Coherent Narrative, Unrelated Narratives) and Retention Interval (Immediate Recall, Delayed Recall). **b** Recall of individual sideplot events: similar to **a**, except recalled details are binned and averaged by Event Number (1, 2) and Coherence, within each Retention Interval group. **c** Recall of main plots: verifiable recalled details for main plot events are summed for each main protagonist, and then binned and averaged by Retention Interval group. Key: Bars = mean recalled details (+/- standard error of the mean), brackets = significant t-tests: +=*p* < 0.10, * = *p* <0.05, ** = *p* <0.01, *** = *p* <0.001

A new group of raters (DL, HR, LFD, TG) was trained to score recall using the same scoring procedure as Experiments 1 and 2. Final interrater reliability was high for sideplot events (mean Pearson’s *r* = .94), and for main plot event totals (mean Pearson’s *r* = .85). As in Experiments 1 and 2, “Either” and “Integrated” details were counted, but not included in analyses of recall performance (see Fig. 4). “Either” details were rare (X̄ = 0.08 details/cue, *SD* = 0.34 details/cue, max = 3.5 details/cue), and “Integrated” details were both rare and only observed for Coherent Narrative events (X̄ = 0.06 details/cue, *SD* = 0.30 details/cue, max = 3.0 details/cue).

#### Data analysis

Statistical methods were generally identical to Experiment 1, except for Bayesian analysis. To further interpret findings from Experiment 3, we used replication Bayes factors (Ly et al., 2019) to quantify the change in evidence following Experiment 1. As detailed by Ly et al. (2019), we first computed a complete Bayes factor by pooling data from both Experiments 1 and 3 (using ttestBF, as described in Experiment 1 Methods). We then divided the complete Bayes factor by the Bayes factor for Experiment 1 alone; this yielded the replication Bayes factor for Experiment 3. This approach to calculating replication Bayes factors is less computationally intensive than approaches which require an approximation of the posterior distribution from a prior experiment; importantly, both approaches can yield identical results (Ly et al., 2019).

### Results and discussion

As with Experiment 1, we focused on differences in recall of sideplot events, and whether this benefit depended on a delay. To test these ideas, we modeled sideplot event recall with a 2 × 2 ANOVA, incorporating a within-subjects factor of Coherence [CN vs. UN] and a between-subjects factor of Retention Interval [Immediate Recall vs. Delayed Recall]. This comparison (see Fig. 4a) revealed a significant main effect of Coherence, *F*(1, 88) = 10.36, η_G_^2^ = .02, *p* = .002. The replication Bayes factor for the main effect of Coherence (BF_10_ = 87.7) suggested that following Experiment 1, Experiment 2 provided an 88-fold increase in evidence in favor of our first hypothesis, that Coherent Narratives would benefit recall of temporally distant events.

However, unlike Experiment 1, there was a significant main effect of Retention Interval, *F*(1, 88) = 15.43, η_G_^2^ = .13, *p* = .0002. Moreover, there was no significant Coherence × Retention Interval interaction, *F*(1, 88) = 0.00, η_G_^2^ < .0001, *p* = .97. In other words, while Experiment 3 replicated a main effect of Coherence from Experiment 1, it failed to replicate the Retention Interval × Coherence interaction. Pairwise contrasts revealed that recall of CN events outperformed UN events in both the Immediate Recall group, *t*(88) = 2.30, *p* = .02, Cohen’s *d* = 0.34, and the Delayed Recall group, *t*(88) = 2.25, *p* = .03, Cohen’s *d* = 0.34, and that recall in the Immediate Recall group outperformed the Delayed Recall group for both CN events, *t*(120) = 3.62, *p* =.0004, Cohen’s *d* = 0.76, and UN events, *t*(120) = 3.59, *p* = .0005, Cohen’s *d* = 0.76. This pattern of findings indicates that there may have been a delay-dependent difference in overall recall (e.g., forgetting); however, the recall benefit for Coherent Narrative events was not dependent on a delay. Furthermore, the replication Bayes factor for the interaction (BF_10_ = 0.11, or BF_01_ = 9.45) suggested that following Experiment 1, Experiment 3 provided a nine-fold increase in evidence in favor of the null hypothesis, that the retention interval had no impact on recall of Coherent Narrative events. These findings suggest that post-encoding memory consolidation was not necessary to facilitate memory for Coherent Narrative events.

To follow up on this result, we investigated the extent to which narrative coherence affected recall separately for the first and second sideplot events (see Fig. 4b). In line with our modeling approach for Experiment 1, within each group, we modeled sideplot event recall using a 2 × 2 ANOVA with within-subjects factors of Coherence [CN vs. UN] and Event Number [1 vs. 2]. Results showed that overall recall was higher for CN than UN events (significant main effect of Coherence) in both the Immediate Recall group, *F*(1, 44) = 6.77, η_G_^2^ = 0.01, *p* = .01, and the Delayed Recall group, *F*(1, 44) = 4.16, η_G_^2^ = 0.01, *p* = .05, and higher for Event 1 than for Event 2 (significant main effect of Event) in both the Immediate Recall group, *F*(1, 44) = 12.19, η_G_^2^ = 0.05, *p* = .001, and the Delayed Recall group, *F*(1, 44) = 22.38, η_G_^2^ = 0.05, *p* < .0001. As in Experiment 2, no significant Coherence × Event Number interaction was observed in either the Immediate Recall group, *F*(1, 44) = 0.24, η_G_^2^ = 0.0006, *p* = .63, or the Delayed Recall group, *F*(1, 44) = 0.01, η_G_^2^ < 0.0001, *p* = .93. Because interactions were not statistically significant, limited inference could be drawn about whether the Coherence benefit was specific to Event 2, or extended to both Events 1 and 2.

Finally, we assessed overall recall differences between groups using main plot recall. Namely, if groups exhibited differing recall performance for main plot events, this might indicate an overall difference in recall ability that could also impact recall of sideplot events. A paired two-sample *t* test revealed that main plot recall was significantly higher for the Immediate Recall group than for the Delayed Recall group (see Fig. 4c), *t*(88) = −2.96, *p* = .004, Cohen’s *d* = 0.67, replication BF_10_ = 1.87.

In summary, results from Experiment 3 generally converged with Experiments 1 and 2 in showing that participants recalled more information about CN events than UN events. That said, although the experimental design was identical, there were important differences in results between Experiment 1 and Experiment 3. In Experiment 1, there were no significant differences in overall recall between groups, whereas in Experiment 3, the Immediate Recall group outperformed the Delayed Recall group at both sideplot and main plot recall. Furthermore, in Experiment 1, the recall benefit for CN events was only evident within the Delayed Recall group, whereas in Experiment 3, this recall benefit was also evident in the Immediate Recall group. These differences in group-specific findings suggest that the Retention Interval manipulation in Experiment 1 may have been confounded by cohort effects. Moreover, the overall pattern of findings suggests that post-encoding memory consolidation was not necessary to enhance retrieval of events that form a coherent narrative. Rather, as proposed by EHM and related theories, this recall benefit is more likely driven by the manner in which narrative events are represented during encoding and retrieval.

## Reanalysis of Experiments 1–3 to control for sentence-level similarity

Whereas EHM and similar theories propose narrative-level mechanisms that determine retrieval, other theories of narrative memory suggest that the degree to which sentences are similar or share certain elements may determine how well they are retrieved (J. R. Anderson & Bower, 1974; Kintsch, 1988, 1992; Reder, 1980). These two sorts of mechanisms are not mutually exclusive. In the current experiments, it was possible that the degree of shared basic content (e.g., words, names, sentence structure) could explain memory for pairs of narrative events in Experiments 1–3. CN and UN side-characters were presented in pairs of events (1 and 2), and it was possible that similarity between the sentences from Event 1 and Event 2 may have driven recall performance. We therefore considered the possibility that the recall difference between CN and UN sideplots could be driven solely by this kind of “lower-level” sentence similarity.

To address this possibility, we first quantified the degree of similarity between sentences in each event in the four stories. We used the freely available, “transformer” version of the Universal Sentence Encoder (USE), a text embedding model designed to convert text into numerical vectors (Cer et al., 2018; see also https://ai.googleblog.com/2018/05/advances-in-semantic-textual-similarity.html). Briefly, the USE uses preweighted layers, previously trained on an expansive textual database, to transform inputted sentences into 512-dimensional embedding vectors that account for words and their respective positions within each sentence. Then, cosine similarity is calculated between each sentence vector, yielding pairwise measures of textual similarity for all inputted sentences. As such, USE similarity serves as a proxy of word-level and sentence-level semantic relatedness for text.

We investigated whether the recall benefit for Coherent Narrative events could be solely explained by sentence-level similarity, or whether narrative coherence enhanced recall over and above any effect of sentence-level similarity assessed by the USE. We calculated USE similarity between sentences from each pair of CN or UN events (8 sentences/Event 1 × 8 sentences/Event 2 = 64 cosine values), and then averaged these values to yield one USE similarity value for each CN or UN event pair (2 CN + 2 UN = 4 USE values per subject). These averaged USE similarity values were entered into a linear mixed-effects model predicting how many details were recalled from all three experiments, using the Afex package in R (https://github.com/singmann/afex; Singmann & Kellen, 2019). We modeled recall of each CN and UN character (summing Events 1 and 2), using the following formula: recall ~ USE similarity + Coherence × Retention Interval + (1|Experiment) + (1|Subject). This approach allowed for individually examining the effects of USE similarity and Coherence, each accounting for the other, and also accounting for how subjects were nested within Retention Interval groups and particular experiments. The model formula was specified by first attempting to fit a maximal random effects structure justified by the experimental design (including random slopes; Barr et al., 2013), followed by systematically pruning the random effects structure until the model converged without a singular solution (i.e., without overfitting; Singmann & Kellen, 2019). As reported above, the only model which converged without a singular solution included only intercepts for random effects. The model fit was quantified using the Akaike information criterion (AIC), and significance for fixed effects was calculated using the Kenward–Roger approximation for degrees of freedom, which is useful for unbalanced mixed designs (Halekoh & Højsgaard, 2014; Singmann & Kellen, 2019).

When accounting for all factors in the same model (AIC = 6686.4), Coherence significantly predicted recall performance, *F*(1, 757.84) = 20.10, *p* < .0001, and USE similarity marginally predicted recall performance, *F*(1, 773.69) = 3.27, *p* = .07; no other fixed effects were significant (*p*s > .22). Interestingly, the marginal effect of USE similarity suggested that, to some degree, sentence-level similarity might predict recall. However, the significant fixed effect of Coherence reflected that even after accounting for sentence-level similarity, the model revealed a positive difference in recalled details between Coherent and Unrelated Narrative events (for the difference, 95% CI [1.03, 2.65]). Furthermore, there was neither a significant effect of Retention Interval, *F*(3, 3.42) = 2.48, *p* = .22, nor a significant Coherence × Retention Interval interaction, *F*(3, 751.45) = 1.24, *p* = .30. These findings suggest that recall is enhanced when separate events can be integrated into a narrative, and that this effect is over and above any variance that could be explained by similarity at the sentence level.

Finally, we investigated whether the Coherence effect was at all driven by the specific content of story events (i.e., “item-level” effects). Although all experiments randomized sideplot event content across subjects, it was still possible that recall differences may have been confounded by which particular events were assigned within each condition (CN vs. UN) for each subject. Therefore, we used the following linear mixed-effects model to test for an effect of Coherence above and beyond any effect of specific event content: recall ~ Coherence × Retention Interval + (1 | Specific Event) + (1 | Experiment) + (1 | Subject). This model only included random intercepts, because more maximal random effects structures (i.e., with random slopes) resulted in singular solutions. When accounting for all factors in the same model (AIC = 11419), Coherence significantly predicted recall performance, *F*(1, 1752.52) = 46.40, *p* < .0001, and no other fixed effects were significant (*p*s > .23). The significant fixed effect of Coherence reflected that even after accounting for item-level effects, the model revealed a positive difference in recalled details between Coherent and Unrelated Narrative events (for the difference, 95% CI [0.82, 1.50]).

## General discussion

The goal of this study was to determine whether recall of separate events would be enhanced if they could be assimilated into a higher-level narrative. Across three experiments, we found that participants recalled more details about temporally distant events that could be integrated into a coherent narrative, relative to events that described unrelated narratives. Results from Experiment 1 suggested that this benefit emerges after a long retention interval, but in the remaining experiments, we found that coherent narratives benefitted recall even at an immediate test (Experiment 3), and we found no evidence that the effect was dependent on sleep-dependent memory consolidation (Experiments 2–3). Below, we consider the implications of these findings for our understanding of the role of semantic information in episodic memory.

Although it is widely accepted that recall benefits from grouping of semantically related items (i.e., “semantic organization” or “semantic elaboration”), contemporary theories of memory often do not describe effects beyond the item level. More recently, researchers have come to appreciate the fact that we actively organize experiences into events, and that event-level organization can influence what and how we remember, over and above what can be accounted for by item features (DuBrow & Davachi, 2013; Franklin et al., 2020; Radvansky & Zacks, 1991; Speer & Zacks, 2005; Swallow et al., 2011). The present results support the idea that episodic memory can be influenced by the extent to which events can be integrated at a higher level. Our results build on a number of studies suggesting that some kind of higher-order organization exists for events in memory (Bartlett, 1932; Bower, 1974; Bower et al., 1979; Cohn-Sheehy & Ranganath, 2017; Greenberg & Verfaellie, 2010; Irish & Piguet, 2013; Kant, 1965; Pichert & Anderson, 1977; Polyn et al., 2009; Radvansky & Zacks, 1991; Rumelhart & Ortony, 1977; Thorndyke & Yekovich, 1980; Tulving, 1972), by demonstrating that these benefits do not depend on temporal contiguity or lower-level features of text.

How does narrative-level organization influence memory? According to EHM (Radvansky, 2012; Radvansky & Zacks, 2017), people infer causal relationships between events during ongoing memory encoding, and these causal links determine which information is more or less accessible during subsequent retrieval (see also Trabasso et al., 1984; Trabasso & Sperry, 1985). The present findings are also compatible with theories proposing that people use schemas to comprehend new information during encoding, and that schemas can subsequently aid retrieval and reconstruction of past events (R. C. Anderson et al., 1983; Mandler & Johnson, 1977; Pichert & Anderson, 1977; Rumelhart, 1977; Rumelhart & Ortony, 1977; Schank & Abelson, 1977; Thorndyke & Yekovich, 1980).

Other theories have conceptualized narratives in terms of networks of associations between overlapping propositions. For instance, Kintsch’s Construction-Integration Model (Kintsch, 1988, 1992) proposes that people form a network of associations between semantically similar items or sentences, and this network of associations is iteratively “pruned” to retain only the strongest semantic associations between narrative events (a “similarity structure”; Kintsch, 1988, 1992). Other accounts (J. R. Anderson & Reder, 1999; Reder, 1980) focus on the idea that overlap between propositions in memory can result in retrieval interference (J. R. Anderson & Bower, 1974; J. R. Anderson & Reder, 1999), but that this interference can be overcome by assimilating these propositions into an overarching concept (Myers et al., 1984; Reder, 1980; Reder & Anderson, 1980; Smith et al., 1978).

The present study was not designed to adjudicate between different accounts of narrative representation per se. The key point in this study is that episodic memory benefits from a narrative-level organization over and above sentence-level or event-level organization. Both Coherent and Unrelated Narratives in the present study had shared content (a recurring side-character), but Coherent Narratives benefitted from the fact that information in each event could be interpreted in the context of information conveyed in the other events. To further rule out the possibility that the memory benefit for coherent narratives was driven by sentence-level or word-level associations, we conducted analyses with the Universal Sentence Encoder (Cer et al., 2018) to quantify sentence-level semantic relationships between events. A mixed-model analysis of data from all three experiments revealed that narrative coherence enhanced recall above and beyond any variance explained by sentence similarity. To the extent that the USE captures textual similarity at the word or sentence level, these findings argue against the idea that the recall benefit for Coherent Narrative events was solely supported by forming associations between conceptually related words or sentences from separated events.

We note that association-based accounts are not entirely inconsistent with accounts of narrative structure. For instance, the idea that a shared concept can reduce retrieval interference among a set of overlapping propositions (e.g., Reder, 1980) is, on its face, similar to the idea that a coherent narrative can reduce interference between discrete events. The main distinction between these ideas is that, in the former case, sentences are considered the basic units of memory (Reder, 1980), whereas in the latter case, events are considered the basic units of memory (Radvansky, 2012). If an association-based account is modified to treat events, and not sentences, as the units of memory (i.e., event–event associations, not sentence–sentence associations), it would potentially predict that integrating two events together would reduce interference in memory. The key point is that memory for naturalistic events relies in part on relationships between events, above and beyond lower-level relationships or associations.

Another goal of the present study was to determine whether the effects of narrative coherence on memory would be altered by post-encoding memory consolidation. Multiple theories suggest that neural processes during sleep, or over a retention interval, tend to integrate related events in memory (Lewis & Durrant, 2011; Moscovitch et al., 2016). Our findings did not provide consistent support for this hypothesis. Although this null result might be seen as a challenge to consolidation theories, it is important to note that several studies have demonstrated that sleep-dependent consolidation can reorganize information in memory (e.g., Antony et al., 2018; Liu & Ranganath, 2019; Petzka et al., 2021; Saletin et al., 2011; Schapiro et al., 2017). For example, Liu and Ranganath (2019) found that sleep-dependent memory consolidation was necessary for temporally distant, semantically related pictures of objects to exhibit retrieval facilitation. A common thread is that all of these studies assessed memory for individual items (e.g., object pictures), rather than meaningful narrative events. It is possible that, lacking an obvious organization (e.g., narratives), inter-item associations in these studies were initially weak (Petzka et al., 2021; Schapiro et al., 2018), such that these items could more easily interfere with each other in memory. If so, these item-based paradigms may have provided more of an opportunity for sleep to strengthen associations (Lewis & Durrant, 2011) or reduce interference (Yonelinas et al., 2019). Conversely, the manner in which narratives are represented during encoding and retrieval may already incorporate strong associations (e.g., causal links; Radvansky, 2012) or minimal interference between events, minimizing any potential role for sleep-dependent consolidation.

If a post-encoding process (e.g., sleep-dependent consolidation) is not necessary to integrate temporally distant events into a larger narrative, then it is worth considering how integration might take place. One possibility is that event integration is supported by some form of reminding during memory encoding (Jacoby, 1974; Ross, 1984; Ross & Bradshaw, 1994). For instance, Kintsch suggested that semantic features of an ongoing event can evoke retrieval of information about a prior event which shares those semantic features (Kintsch, 1988, 1992). According to Kintsch, this kind of reminder-evoked retrieval would specifically take place for events that form a larger narrative, and would support their integration during memory encoding (Kintsch, 1988, 1992). More broadly, recent experiments and computational models have suggested that some form of memory retrieval shapes ongoing encoding of complex events (Franklin et al., 2020; Lu et al., 2020; Stawarczyk et al., 2020; Wahlheim & Zacks, 2019).

An alternative possibility is that different events might be initially encoded as distinct memories (e.g., Chanales et al., 2017; Kumaran, 2012), and that integration across temporally separated events might occur as events are reconstructed during memory retrieval (e.g., Kumaran, 2012). The current behavioral study was not designed to discern between encoding-phase and retrieval-phase processes for integration. Some neuroimaging studies have suggested that the human brain can support integration during either memory encoding or subsequent retrieval (e.g., Horner et al., 2015; Zeithamova & Preston, 2010). These studies have generally not investigated the role of narrative coherence in memory integration. As we report elsewhere (Cohn-Sheehy et al., 2020), our recent neuroimaging work suggests that coherent narrative events might become integrated during memory encoding.

Interestingly, in addition to the consistent recall benefit for CN events, we consistently observed that participants recalled more details about Event 1 than Event 2 for each CN and UN character. Although we did not initially predict this effect, it is possible that, as proposed in EHM, this difference might reflect some kind of retrieval interference between discrete events in memory (Radvansky, 2012). The present experiments were not designed to assess this possibility; however, future experiments could adapt our approach to assess recall one event at a time (i.e., separately cueing Event 1 or Event 2 for each character). For instance, EHM might predict that cueing either CN Event 1 or CN Event 2 would result in retrieval facilitation when subsequently cueing the other event. However, for a UN character, cueing either Event 1 or Event 2 might instead result in retrieval interference when subsequently cueing the other event.

One challenge in studying memory for complex events and narratives is that there are often many uncontrolled variables or effects that might be idiosyncratic to particular stimuli. The present study was therefore designed to control for several possible confounds. By randomizing event content across subjects, the stimuli minimized any content-driven influences on memory, enabling a direct investigation of narrative structure and memory. Moreover, the use of a mixed effects model confirmed that our findings were not solely explained by stimulus content. Furthermore, there were several controls for attention and perception. Stimuli were presented binaurally and at a fixed rate and volume, minimizing any differences in initial perception of the stimuli. Additionally, sideplot events were embedded, minutes apart, within main protagonist stories, such that participants were more likely to pay attention to main plots of these stories, not sideplot events. Because these controls were implemented, it is reasonable to suggest that recall differences were not driven by more easily sustained attention to events that formed a coherent narrative.

The scoring approach in this study was aimed at assessing overall recall of events; however, it might be useful for future studies to subcategorize recalled details. For instance, some theories differentiate between recall of “perceptual” details and “gist” information (e.g., Moscovitch et al., 2016), and this distinction is incorporated in the scoring protocol for the Autobiographical Memory Interview (Levine et al., 2002). This approach would not have been useful in the present study because, although the stories included sensory details (e.g., “purple scarf”), these details were conveyed in a relatively abstract form through spoken language. Thus, we would not expect a meaningful distinction between recall of sensory details and gist information in this study. However, such an approach could be used in studies of recall for multimodal stimuli such as films, which incorporate perceptual information that is not limited to spoken language. Alternatively, recognition-based assessments of narrative memory can discern between different kinds of information in memory (e.g., surface form, textbase, event model; van Dijk & Kintsch, 1983; Fisher & Radvansky, 2018; Long et al., 2012).

In closing, our work shows that recall of temporally distant events can be enhanced if events can be integrated into a coherent narrative, which suggests that narratives provide a beneficial organization for events in memory. Ongoing neuroimaging investigations will further reveal the narrative architecture that supports episodic memory (Cohn-Sheehy et al., 2020).

## Supplementary Information

ESM 1 (PDF 93 kb)

ESM 2 (PDF 161 kb)

ESM 3 (PDF 130 kb)

ESM 4 (PDF 86 kb)

## Data Availability

The materials for this study have been made available as supplemental files. Upon publication, data, and analysis scripts will be made available through the Open Science Framework: https://osf.io/uw4an/?view_only=d6b7d6de438c472e86f348630e730e86
