# Supplementary material for: Narratives bridge the divide between distant events in episodic memory
Source: Mem Cognit. 2021 Apr 26;50(3):478–94. doi: 10.3758/s13421-021-01178-x (PMC8546012; doi:10.3758/s13421-021-01178-x)
Supplement: Supplementary file 1 — (PDF 93 kb) [file 13421_2021_1178_MOESM1_ESM.pdf]

Supplemental Methods

Stimulus creation

All sentences were designed to fit within 5 seconds each. This involved both altering the words and syntax of sentence text as well as editing recorded audio. BICS recorded all sentences, employing a cardioid condenser microphone (Shure, Inc.) and Mbox interface (Digidesign, Inc.) and speaking in a manner which attempted to minimize any differences in emphasis or tone of voice between sentences. Recorded audio was mixed using Pro Tools 8 LE software (Avid, Inc.). As needed, audio for individual sentences was cropped, removing extra time and/or loud breaths, and artificially lengthened or compressed using "elastic audio" to fit each sentence within 5 seconds. Audio was then filtered with a high pass at 80 Hz and low pass at 16kHz, peaks were normalized within and across clips, and finally a compressor was applied to all audio to maximize loudness without a tradeoff in subjective clarity. This approach was undertaken in order to minimize low-level differences in audio across all recorded sentences, and to enable smooth transitions between sentences to be perceived as continuous.

By standardizing all sentences at 5 seconds each, we were able to equalize the length of audio for all stories and all events. Each of the four stories contains 48 sentences at 5 seconds each, and therefore each story elapses for 4 minutes. Within each story, each sideplot event contains 8 sentences (40s each), and these sideplot events are bookended by 8 sentences of the main protagonist story which do not involve one of the four side-characters of interest (40s each). There are therefore 16 main protagonist sentences placed between two sideplot events, however, this is comprised of two separate main protagonist events, because there is a temporal shift in the 9th of these sentences (e.g. "twenty minutes later," "one hour later"). Previous research has demonstrated that temporal shifts are associated with the perception of boundaries

which segment events in perception and in memory (Zacks et al., 2007, 2009). Pilot experiments conducted in an independent sample (data not shown) using two alternate sideplot arrangements demonstrated that participants reliably perceive an event boundary at these temporal shifts within our constructed stories, as well as at the starts and ends of side character appearances (black bars in Figure 1). Therefore, we presume that each sideplot event is a distinct *event* surrounded by two main protagonist events, and that there are two main protagonist events which separate two sideplot events present within a given story. This was in line with our intent to minimize any potential for interactions between sideplot events within a given story. In all, main protagonist and sideplot events are distinct events of equal length (8 sentences/40 seconds each), and the quality and timing of audio clips should not be involved in any observed effects.

Familiarization tasks

Three brief tasks aimed to familiarize participants with ten characters from the stories, in order to increase the likelihood that character names and relationships could be used as successful recall cues. Two characters were the main protagonists (Charles, Karen), four characters were CN or UN side-characters (Beatrice, Melvin, Sandra, Johnny), and four additional characters had supporting roles within the main protagonist stories (i.e. main plots, not sideplots). Participants were instructed to “get to know these characters,” because they would appear in the upcoming stories. In the first task, character names were presented alongside faces (Bainbridge et al., 2013) that were selected for high memorability and diversity. Each of 10 name-face pairs appeared on the center of a black background for 4 seconds, followed by a 1s fixation screen, and were repeated for a total of 20 trials. In the second task, participants were presented with character-character relationships for the same ten characters, and were instructed to familiarize themselves with these relationships. For each of 20 trials (2 repetitions per pair, 4s

per trial, 1s fixation), character names and faces were presented side-by-side, with their relationship displayed on the right side of the screen (e.g. "best friend"). The third task was identical to the second task, except there was a response component. On 20/30 trials, relationships were correct, whereas on 10/30 trials, relationships were incorrect. Participants were required to indicate whether the relationship was correct or not (3s response window), after which they were shown the correct relationship (2s), which was displayed in green if previously correct, or in red if previously incorrect. In all, these tasks were designed to orient participants' attention to characters, their relations, and the potential for these characters to be involved in situations within the upcoming stories, with the aim of successfully evoking subsequent recall by cuing with character names.

Sleep questionnaires (Experiment 2 only)

Standard sleep questionnaires were collected from Experiment 2 participants (Broughton et al., 1982; Hoddes et al., 1972, 1973; Horne & Ostberg, 1976; Johns, 1991; Terman & Terman, 2005). The Epworth Sleepiness Scale (ESS), Morningness-Eveningness Questionnaire (MEQ), and the Stanford Sleepiness Scale (SSS) were administered at the beginning or end of each session (i.e., before or after stimulus presentation or recall). The SSS was administered before and after encoding (SSS-Encoding) and before and after retrieval (SSS-Retrieval). Participants were additionally asked to report hours of sleep for three nights preceding the experiment. As depicted in **Table S1**, none of these measures significantly differed between Wake and Sleep groups in Experiment 2.

Epworth Sleepiness Scale (ESS). The ESS assesses daytime sleepiness by asking participants to rate, on a 4-point scale, their usual chances of dozing off or falling asleep while engaged in eight different activities (e.g., watching television, sitting, lying down, etc.). It ranges

from 0 to 24, with higher numbers indicating higher daytime sleepiness.

Morningness-Eveningness Questionnaire (MEQ): Consisting of 19 items, the MEQ assesses individual differences in morningness and eveningness—the degree to which participants are active and alert at certain times of day. Scale items query preferences in sleep and waking times, and subjective “peak” times at which respondents feel their best.

Stanford Sleepiness Scale (SSS): The SSS evaluates sleepiness at specific moments in time. Consisting of only one item, the scale requires respondents to select one of seven statements best representing their level of perceived sleepiness. This was administered before and after encoding, and before and after retrieval—**Table S1** presents the averages across these two sets of measurements (SSS (Encoding), SSS (Retrieval)).

Average Hours of Sleep: This is the mean daily sleep reported by participants for the three days prior to experiment Session 1.

Hours of Sleep the Night Before: This is the amount of sleep participants reported for the night before Session 1 of the experiments.

Table S1

Sleep questionnaire results for Experiment 2

Sleep Questionnaire	Wake	Sleep
**ESS**	7.40 (3.29)	8.18 (3.63)
**MEQ**	46.51 (7.60)	47.22 (8.19)
**SSS (Encoding)**	2.97 (0.90)	2.76 (1.32)
**SSS (Retrieval)**	2.95 (0.92)	2.76 (1.32)
**Average Hours of Sleep**	7.21 (1.01)	7.30 (1.01)
**Hours of Sleep the Night Before**	6.33 (0.99)	7.09 (1.28)

Note. See Supplemental Methods (above) for more information on measures. No significant differences were found between Wake and Sleep groups.

Supplemental Results

Outlier analysis (Experiment 1 only)

As described in the **Experiment 1 Results**, four subjects within the Delayed Recall group were identified as outliers in analyses of main plot recall. We investigated the impact of these subjects on sideplot recall findings. On average, sideplot recall in these subjects (Coherent Narratives: $\bar{X}$=15.7 details/cue, SD=7.9 details/cue, min=4.0 details/cue, max=27.5 details/cue; Unrelated Narratives: $\bar{X}$=13.1 details/cue, SD=7.6 details/cue, min = 4.5 details/cue, max=25.5 details/cue) was numerically higher than for other Delayed Recall subjects (Coherent Narratives: $\bar{X}$=10.4 details/cue, SD=9.3 details/cue, min=0 details/cue, max=32.0 details/cue; Unrelated Narratives: $\bar{X}$=7.3 details/cue, SD=7.1 details/cue, min = 0 details/cue, max=31.5 details/cue).

Therefore, we tested whether removing these outlier subjects would change the pattern of findings for sideplot recall. We performed a 2 x 2 ANOVA incorporating a within-subjects factor of Coherence [CN vs UN] and a between-subjects factor of Retention Interval [Immediate Recall vs Delayed Recall]. Even after removing these outlier subjects, the ANOVA revealed a significant main effect of Coherence (F(1,66)=8.49, η_G^2=.017, p=.005) which was qualified by a significant Coherence X Retention Interval interaction (F(1,66)=6.32, η_G^2=.013, p=.014), and no significant main effect of Retention Interval (F(1,66)=1.89, η_G^2=.024, p=.17). The significant interaction reflected that within the Delayed Recall group, CN events were recalled in greater detail than UN events (t(66.0)=3.73, p=0.0004, Cohen's d=0.66). Furthermore, recall of CN events within the Delayed Recall group was significantly higher than recall of either CN events (t(86.7)=2.21, p=0.03, Cohen's d=0.54) or UN events (t(86.7)=2.36, p=0.02, Cohen's d=0.57) within the Immediate Recall group. Thus, removing main plot outlier subjects from the sideplot recall analysis did not change the overall pattern of findings.

Supplemental References

Bainbridge, W. A., Isola, P., & Oliva, A. (2013). The intrinsic memorability of face photographs. Journal of Experimental Psychology: General, 142(4), 1323–1334. https://doi.org/10.1037/a0033872

Broughton, R. (1982). Performance and evoked potential measures of various states of daytime sleepiness. Sleep, 5 , S135-S146.

Hoddes, E., Dement, W., & Zarcone, V. (1972). The development and use of the Stanford sleepiness scale (SSS). Psychophysiology, 9 , 150.

Hoddes, E., Zarcone, V., Smythe, H., Phillips, R., & Dement, W.C. (1973). Quantification of sleepiness: a new approach. Psychophysiology, 10 , 431–436.

Horne JA and Östberg O. A self-assessment questionnaire to determine morningness-eveningness in human circadian rhythms. International Journal of Chronobiology, 1976: 4, 97-100.

Johns MW. A new method for measuring daytime sleepiness: The Epworth Sleepiness Scale. Sleep 1991; 14(6):540-5.

Terman M, Terman JS. Light therapy for seasonal and nonseasonal depression: efficacy, protocol, safety, and side effects. CNS Spectrums, 2005;10:647-663.

Zacks, J. M., Speer, N. K., Swallow, K. M., Braver, T. S., & Reynolds, J. R. (2007). Event perception: A mind-brain perspective. Psychological Bulletin, 133(2), 273–293. https://doi.org/10.1037/0033-2909.133.2.273

Zacks, J. M., Speer, N. K., & Reynolds, J. R. (2009). Segmentation in reading and film comprehension. Journal of Experimental Psychology. General, 138(2), 307–327. https://doi.org/10.1037/a0015305
