# Supplementary material for: Narratives bridge the divide between distant events in episodic memory
Source: Mem Cognit. 2021 Apr 26;50(3):478–94. doi: 10.3758/s13421-021-01178-x (PMC8546012; doi:10.3758/s13421-021-01178-x)
Supplement: Supplementary file 2 — (PDF 161 kb) [file 13421_2021_1178_MOESM2_ESM.pdf]

Supplementary Data 1: Side-character events and main protagonist stories.

Note: *Possible side-character events are indexed in terms of the first versus second appearance for a given side-character (1 vs 2) and sideplot version (A vs B).*

Events involving recurring side-characters (i.e. "sideplot" events)

Beatrice 1A

A tremor ran through his leg, as his phone received a call from his cousin Beatrice Small.
"Sorry to bug you this early," Beatrice said anxiously, "But, I'm having a bit of a dilemma."
"My girlfriend and I are having trouble agreeing on whether to adopt a lab or a corgi."
"Is this really that important?" Charles grumbled, "I don't know anything about dogs."
"It's been rocky the past week," she replied, "And she thought a dog would make things easier."
"But if she insists on adopting a labrador with a pink collar, I'm moving out!"
"Why don't you go to the animal shelter together," Charles suggested, "And try to agree there?"
"That's a great idea, thanks for the advice!" replied Beatrice before hanging up.

Beatrice 2A

Just then Karen's neighbor Beatrice Small stopped by their table, looking downcast.
Karen noticed a crumpled tissue in her right hand and asked, "Beatrice, are you okay?"
"Not really," Beatrice muttered, "This is one of the worst days I've had in a while."
"When I came home this labrador in a pink collar was barking behind my front door."
"And now," Beatrice continued, "I have to find another place to live, and soon!"
"That sounds awful," said Karen, "Is there anything I can do to help with the process?"
"Sure," said Beatrice, "If you know a one-bedroom apartment, let me know!"
"Or any good looking singles," Beatrice quipped, winking as she left their table.

Beatrice 1B

A tremor ran through his leg, as his phone received a call from his cousin Beatrice Small.
"I know you're probably busy," Beatrice said frantically, "But, I'm up against a deadline."
"I need to raise at least a hundred dollars to bike in the marathon an hour from now."

“And you think I have that kind of money?” Charles grumbled, “I’m not that rich.”
“I’m fighting the pain,” she replied, “I was diagnosed with seronegative arthritis.”
“My doctor said exercise helps the pain, so I want to test that to the extreme!”
“What if I donate fifty dollars to your cause,” Charles offered, “Would that be enough?”
“That would be fantastic, thanks so much!” replied Beatrice before hanging up.

Beatrice 2B

Just then Karen’s neighbor Beatrice Small stopped by their table, smiling ecstatically.
Karen noticed a concerning cast on her left arm and asked, “Beatrice, are you okay?”
“Absolutely!” Beatrice chimed in, “It could have been a lot worse than this.”
“My bicycle skidded out on a damp road, and I broke my fall with this arm.”
“But then,” Beatrice continued, “I got back up and biked all the way back!”
“That’s incredible,” Karen replied, “But wasn’t it painful to keep using that arm?”
“Oh sure,” said Beatrice, “Though this is the least pain I’ve ever felt in my life!”
“You should bike with me some time,” Beatrice suggested as she left their table.

Melvin 1A

There was a tap on Charles’ shoulder from his neighbor Melvin Doyle, wearing an Elizabethan collar.
“Check out this old collar, Charles, I’m just on the way to my big audition this morning.”
“Can you guess which play it’s for,” Melvin quizzed, but Charles just shook his head.
“It’s Shakespeare’s Hamlet, why else would I go around wearing this ridiculous collar?”
“They’re short on actors, so if they like me, I could end up playing Hamlet or Ophelia.”
“I had to prepare two monologues, including Hamlet’s, ‘to be or not to be’ speech.”
“I’ll do Ophelia’s speech in a falsetto, ‘O what a noble mind is over thrown’.”
Melvin patted Charles hard on the back, saying “Wish me luck!” as he left.

Melvin 2A

Her conversation with the driver was cut off by a phone call from her friend Melvin Doyle.
“Hey Karen,” said Melvin, “Do you know what you’re doing a month from tonight?”

“I’m not yet sure,” she replied, “Why, is there something important next month?”
“Only yours truly taking the stage for a one night performance,” he replied jubilantly.
“That’s fantastic,” said Karen, “I never doubted for a moment they’d let you perform!”
Melvin blurted in a high falsetto, “Oh what a noble mind is overthrown!”
He returned to his normal voice, proclaiming, “You’ll be hearing more of that in a month!”
“I’ll keep you posted on the date and tickets, so until then, wish me luck!”

Melvin 1B

There was a tap on Charles’ shoulder from his neighbor Melvin Doyle, wearing a soiled white coat.
“See this new coat, Charles, I ruined it diving into the dumpster this morning!”
“I bet that sounds really weird,” Melvin suggested, and Charles nodded in assent.
“Well I thought I dropping my keys in there, but now I think it was the other dumpster.”
“So now I’m locked out of my house, and there’s this Sicilian pizza I have in the oven.”
“If I happen to relocate my keys in time, it might become the best dish I’ve ever made!”
“But I did leave the oven on, so I should probably run back there as soon as possible.”
Melvin suddenly grimaced at Charles, blurting, “Oops!” as he sprinted away.

Melvin 2B

Her conversation with the driver was cut off by a phone call from her friend Melvin Doyle.
“Hey Karen,” said Melvin, “Do you happen to know a good house-cleaning service in town?”
“I know a couple housekeepers,” she replied, “What exactly do you need to get cleaned?”
“Only a fully charred, ashy kitchen from a grease fire,” he replied ironically.
“Oh no,” said Karen, “I thought you told me you’ve never even used your kitchen.”
Melvin exclaimed, “Thank goodness the fire department has a spare key to my place.”
He continued softly, “By the way, I found out burnt cheese actually tastes delicious.”
“Anyways, I’d appreciate you sending along the cleaners’ names when you get home!”

Sandra 1A

Charles was saved by a call from his friend Sandra Mayes, who sounded anxious.

“I have a blind date tomorrow afternoon,” she began, “And I’m getting really nervous.”
“I met this guy online, who’s really funny, and witty, but I don’t know what he looks like.”
“Isn’t that the point of a blind date,” Charles began, though he was interrupted by Sandra.
“He might be the guy who broke my friend’s heart, this smooth-talking womanizer type.”
“Well, that would be interesting,” said Charles, “You want some kind of advice from me?”
“No,” replied Sandra, “I just wanted someone to talk to, it’s a bit nerve-wracking.”
“But if he’s the guy with long hair, sideburns, and a leather jacket, he’ll be sorry.”

Sandra 2A

As Karen sought out a seat, she noticed her yoga partner Sandra Mayes waving from a booth.
“Didn’t expect to see you,” said Sandra, “I was hoping it would be a man arriving.”
“Don’t tell me another date is standing you up this time,” said Karen.
“Well he’s over twenty minutes late, and I’ve yet to get a text from him,” said Sandra.
“Sandra, why are you wasting your time, you deserve much better,” said Karen.
“I guess you’re right,” sighed Sandra, and she slowly stood up from the booth.
As she was leaving, in walked a long-haired man with sideburns and a leather jacket.
Sandra slapped him across the face and walked out, leaving him in shock.

Sandra 1B

Charles was saved by a call from his friend Sandra Mayes, who sounded stressed.
“My first mural show starts tonight,” she began, “And a lot of sponsors are coming.”
“I get headaches from dealing with just a few people, let alone a hundred philanthropists!”
“Aren’t they supposed to purchase your art,” Charles began, but Sandra sighed.
“The short critic with the red sportscoat hates murals, and they always listen to her.”
“Don’t worry about her,” said Charles, “But do you want me to come and keep you company?”
“Yes please,” replied Sandra, “I’ll be less anxious if you keep her from talking to me.”
“See when she actually likes artists, she starts obsessively following them around!”

Sandra 2B

As Karen sought out a seat, she noticed her yoga partner Sandra Mayes hiding in a booth.

"Thank goodness," said Sandra, "I was worried you would be someone I met last night."
"Are you telling me that you had way too much of a good time at the show?" said Karen.
"Actually a dozen philanthropists are hocking me to paint their mansions," said Sandra.
"Then what are you hiding from Sandra, you could make a fortune off them," said Karen.
"There's just too many of them," Sandra muttered, massaging her temples with both hands.
As she swallowed an Advil, a short woman in a red sportscoat entered the restaurant.
Sandra ducked below the table while the woman scanned the restaurant, until she left.

Johnny 1A

As Charles turned abruptly to take a seat he nearly collided with his coworker Johnny Pratt.
"Sorry man," said Johnny, rummaging around the fading blue carpet and waiting chairs.
"Hey Charles," he said, "Have you seen my mashed potato recipe lying around here?"
"No I haven't," said Charles, "And what are you doing with a mashed potato recipe?"
"It's a family recipe, and my grandmother wrote out only one copy for me," he replied.
"You see," Johnny continued, "My boyfriend's real upset that I've been so unavailable lately."
"He said I could make it up to him if I made my grandmother's mashed potatoes for dinner."
"But if I don't, he might move out," Johnny explained, "So I better find the recipe!"

Johnny 2A

As the line shuffled forward, Karen was surprised by a text from her brother-in-law Johnny Pratt.
"Karen, any chance you know a cheap airline to Hawaii?" read Johnny's text.
She replied, "No, but tell me, is there some special occasion you're celebrating?"
"Not exactly," he responded, "My boyfriend just wants to do something nice for me."
"He thought that Hawaii would be a great place to relax and have fun," he continued.
"He's sorry for being too tough on me recently, and now he wants to make up for it."
"Also," Johnny added in a subsequent text, "You want some leftover potatoes?"
"Of course," Karen replied, "You know how much I always look forward to your cooking."

Johnny 1B

As Charles turned abruptly to take a seat he nearly collided with his coworker Johnny Pratt.

“Sorry man,” said Johnny, who was searching frantically behind chairs and tables.

“Hey Charles, he said, “Any chance you’ve seen my American Express card lying nearby?”

“Actually I haven’t,” said Charles, “And how on earth did you lose that around the office?”

“It’s a bit embarrassing, I forgot that my mortgage payment was due earlier today.”

“You see,” Johnny continued, “The payment was late, but the bank gave me an extension.”

“They said that if I used my credit card they’d let me make the next payment by this morning.”

“If I don’t pay before they close, I’ll lose everything,” Johnny explained, “So I better find it!”

Johnny 2B

As the line shuffled forward, Karen was surprised by a text from her brother-in-law Johnny Pratt.

“Karen, any chance you know a lawyer who does financial cases?” read Johnny’s text.

She replied, “No, but don’t tell me you got yourself into trouble again.”

“Not exactly,” he responded, “Though maybe I could have been more careful.”

“The bank’s coming after me, and I really could use representation,” he continued.

“But, they’d have to be okay with no payment up front, since I’m now broke.”

“Also,” Johnny added in a subsequent text, “Could I stay at your place for a while?”

“Of course,” Karen replied, “You know you can use my couch if need be.”

Main protagonist stories (i.e. “main plot” events)

Charles 1 (aka Story 1)

1A: It was Charles Bort’s big break: there was finally a scandal he could photograph.
For weeks local journalists had been hounding Mayor Frank about his abuse of power.
Now, Charles would have his turn, to photograph the Mayor as he responded to impeachment.
Early Tuesday morning Charles shivered in the grey-walled newsroom wearing his purple scarf.
His boss was due any minute with word of where and when the Mayor could be photographed.
Charles impatiently devoured many spoonfuls of Greek yogurt behind the piles on his desk.
Photos were bursting from manila folders and weeks of newspapers with post-its.
Charles studied each photo, the angles too forgiving, the Mayor smiling too much.

[BEATRICE 1A or 1B]

1C: The newsroom was quiet, but Charles could make out footsteps approaching from the echoing hallway.
Charles knew those heavy steps, as they belonged to the managing editor, Charles’ boss.
The wooden door creaked open, and Charles called out, “What’s doing, boss?”
“Don’t get cozy, you got a date with Mayor Frank’s press conference in twenty minutes.”
“Twenty minutes?!” Charles yelled, “How the hell am I getting to City Hall in twenty minutes?”
“Take my bicycle,” said the managing editor nonchalantly, “It’s in the mailroom.”
Charles scrambled to pack his Nikon camera, detachable flash, and extra batteries.
As Charles ran to the exit, his boss yelled, “Don’t forget to send me the photo!”

1D: Twenty minutes later Charles chained the bike to a meter by the marble City Hall steps.
A crowd had formed on the steps around the mayor’s podium, but Charles could not see the mayor.
He needed a better vantage point, so he maneuvered all the way to the front of the crowd.
Since the mayor was late, the other local reporters and photographers stood around chatting.
A woman with a small camera and notepad stood next to Charles, scribbling away.

Charles got her attention, and he asked, “Any idea why Mayor Frank is running late?”
She chuckled, “Apparently he dropped his daughter off at school, though he never does that.”
“I guess he wants us to go easy,” Charles laughed heartily, “But too bad he’s a crook!”

[MELVIN 1A or 1B]

1F: Charles compulsively checked his watch, expecting the Mayor to arrive at any moment.
He adjusted the lighting and focus on his camera, aiming it above the podium microphone.
He would have to catch the Mayor off-guard to capture his real, non-rehearsed facial expressions.
The crowd grew silent as the rumble of a police motorcycle approached, then cut its engine.
It escorted a limousine from which Mayor Frank emerged with two security guards.
As soon as the Mayor reached the podium one journalist yelled, “Why did you graffiti the courthouse?”
The Mayor replied, but seeing his opportunity Charles exclaimed “Smile, Mr. Mayor.”
The mayor glared at the camera, providing Charles the photograph that could make his career.

<u>Charles 2 (aka Story 2)</u>

2A: Charles Bort ate lunch with his mother Ethel, but he obsessed about an overdue promotion.
Tuesday at noon they sat on a green park bench, Charles sweating under his beret.
Ethel handed Charles half of her tuna salad sandwich as she took a bite of her own.
“Sorry I missed last week, mom, I’ve been trying to build my portfolio,” said Charles.
“Don’t worry about it,” Ethel replied calmly, “You know I’m very proud of you.”
“But Charles,” she continued, “Can you at least tell me you’re enjoying yourself these days?”
“That’s irrelevant, I’ll be more satisfied if I finally get promoted!” he replied.
“Come on,” said Ethel, “Charles, isn’t there some special person in your life?”

[SANDRA 1A or 1B]

2C: Charles put away his phone, and he asked his mother if she had seen his photograph.
“I don’t understand why they would take a photograph of a photographer,” Ethel mused.
“Don’t be silly, mom, the front page photo I took of the Mayor!” Charles chided.
“You sent me that article link,” she explained, “But there wasn’t any photograph in it.”
“That can’t be,” he replied, “The photo is supposed to tie the whole report together!”
Charles waited for the article to slowly load on his smartphone, clenching his teeth.
When the article finally appeared, Ethel complained, “I told you there was no photo!”
Charles cried, “But that photo’s my chance at becoming photography editor!”

2D: An hour later, Charles walked quickly up the escalator to the newspaper’s administrative office.
He traversed several aisles of grey-walled cubicles to the managing editor’s closed door.
“He’s in a meeting,” explained the secretary at the long desk adjacent to the door.
“But it’s urgent,” said Charles, “It’s about today’s paper, he needs to hear it now.”
“It’s a bit late for that,” replied the secretary, who held out a fresh print of the evening paper.
Charles took hold of it and scanned the photo of a smiling mayor on the front cover.
“This is wrong!” he shouted, “I need to speak to him now, it’s the wrong photo!”
“Look Charles, just take a seat over there, and I’ll let you know when he’s ready.”

[JOHNNY 1A or 1B]

2F: Just then the managing editor’s door swung open, and the secretary waved Charles in.
The managing editor sat in a tall wooden chair at his mahogany desk, smoking a pipe.
“What can I do for you, Charlie-boy,” he asked, not looking up from his stack of paperwork.
“There’s a big problem,” Charles said gravely, “You got the wrong photograph on the cover!”
“Oh, you mean the photograph we had to buy since you didn’t do your job,” he replied.
“No way,” said Charles, “I sent you the perfect picture of the mayor this morning!”
Charles suddenly realized he had never pressed “Send” on the email, and his boss was right.
“Never mind,” Charles relented, and left to sulk at his desk, with no promotion.

Karen 1 (aka Story 3)

1A: Karen Joyce was done with culinary school, jobless, and figuring out her life.

Late Tuesday evening she was on her way to a long overdue coffee date with her best friend Will.

She sat in the heated leather backseat of a taxi wearing her favorite silver earrings.

“Any idea how much longer it’ll take to get to Café Trieste,” she asked the driver.

“Maybe another ten minutes, why, are you in a hurry?” replied the driver.

“I guess I’m just excited,” said Karen, “My best friend says he has good news for me.”

“And since apparently it’s impossible to be employed as a chef, I’d love any news!”

“You know, this person I picked up yesterday was just laid off from a restaurant,” added the driver.

[MELVIN 2A or 2B]

1C: Karen flipped through email on her smartphone to see if any job applications came through.

There was one unread message in her inbox, but it was only spam from a dating website.

“Hey, how hard is it to live off a taxi driver’s salary?” she inquired of the driver.

“You don’t want to know,” the driver replied, “You’d be much better off as a chef.”

“You’re probably right,” said Karen, “But it looks like I need to keep my options open.”

The driver said, “Despite my PhD I’ve been a bouncer, barkeep, and driver.”

“If I were you,” the driver continued, “I’d do anything but those three jobs!”

Karen decided the driver deserved a nice, large tip when they arrived at the cafe.

1D: Fifteen minutes later Karen walked through the café entrance, and her smartphone buzzed.

“First table on the right, outdoor back patio,” read the just-received text from her best friend.

She found Will at that exact location, in a black metal chair under a tall space heater.

“Karen, it’s been too long!” he exclaimed, standing up to give her a tight embrace.

As he pulled out a chair for her to sit, he inquired, “What’s new with you?”

“Come on Will, you know the answer,” she chuckled, “Absolutely nothing!”

“You mean, you’re not yet the rich, world renowned Chef Karen?” he joked.

“Too true,” she replied half-sadly, “Tell me, what’s going on in your life?”

[BEATRICE 2A or 2B]

1F: A waiter came by to take their orders for two large hot chocolates and cheesecake.

“So Will,” Karen began, “What’s this good news you were gonna tell me?”

“Right,” he said, “You ever heard of this place, Chez Claire, on 8th Avenue?”

“Of course, that’s the French place where you had your engagement party!” she replied.

“Well, I happen to be good friends with the manager, he’s a nice guy,” Will began.

He paused to let the waiter place heaping mugs of hot chocolate in front of them.

Will continued, “He says they could use an extra cook, and it’s all yours if you want it.”

Karen exclaimed, “I’ll take it!” clinking her mug against Will’s in celebration.

<u>Karen 2 (aka Story 4)</u>

2A: Karen Joyce was on edge, already running late for the one cooking job she could find. Mid-Wednesday afternoon she waited impatiently at the city tow lot to retrieve her impounded car.

Earlier she had parked her car illegally to make a quick errand, only to discover it missing.

Now she waited in the long, slowly-moving line, shivering in a blue apron over her clothes.

There was only one lot attendant at a kiosk by the chain link fence, taking his time.

Karen kept checking the time on her smartphone, wishing the attendant would speed things up.

However it became clear that the man at the front of line, yelling louder and louder, was the problem.

The next two in line yelled at this man, imploring him to finish up so they could get their cars.

[JOHNNY 2A or 2B]

2C: Since a second lot attendant had arrived, the line was moving faster, and Karen was next.

“Look, I know you’re busy,” she began, “But I have an important interview and I need my car.”

“You and everyone else,” replied the attendant snidely, “But now, which car is it?”

“The black Honda Accord, which you probably towed from 4th and Main earlier today.”

He ran his finger over the handwritten table of vehicles, and stopped suddenly at one line.

“Don’t know how to best say this, ma’am, but your car blew up on the way over here.”

“You’ve got to be kidding me,” she muttered under her breath, frozen in shock.

“No joke,” he replied, “And there’s a few forms for you to fill out right over here.”

2D: An hour later, Karen sprinted from the local bus stop and through the open door of the French restaurant.

She stopped out of breath in front of the cash register, scanning the red-leather interior.

She nearly jumped when the restaurant manager stood up from behind the register.

“Sorry for the scare, I’m fixing the credit card mechanism,” he stated calmly.

“It’s okay,” she replied anxiously, “I’m running late, I’m interviewing for the chef opening.”

“Ah Karen,” he said cheerily, “I bet Executive Chef Renee can’t wait to meet you.”

“That’s me,” she replied, “And if my car hadn’t been towed I’d have been here nice and early.”

“Why don’t you have a seat,” he offered, “and I’ll go on back to let her know you’re here.”

[SANDRA 2A or 2B]

2F: The manager appeared from behind swinging double doors, and motioned Karen over.

Behind the doors was a steel-countered kitchen, where a couple cooks were chopping vegetables.

Karen leaned back against the counter, and took a couple deep breaths to calm her nerves.

Just then, Executive Chef Renee burst into the kitchen, her face flushing purple.

“What gives you the right to show up late to a job in my kitchen?” shouted Renee.

“I’m sorry, my car exploded, it was out of my control,” apologized Karen.

“No, no apology is acceptable, get the hell out of my kitchen!” replied Renee.

Karen passionately displayed the back of her middle finger to Renee, and walked out.
