# Supplementary material for: Narratives bridge the divide between distant events in episodic memory
Source: Mem Cognit. 2021 Apr 26;50(3):478–94. doi: 10.3758/s13421-021-01178-x (PMC8546012; doi:10.3758/s13421-021-01178-x)
Supplement: Supplementary file 3 — (PDF 130 kb) [file 13421_2021_1178_MOESM3_ESM.pdf]

Supplementary Data 2: Recall scoring instructions

1. **Open participant transcript in Microsoft Word.**
    a. Save as [Your initials]_annotated_[transcript filename}
        i. E.g. BICS_annotated_Sub06_v05.docx
    b. Enable Track Changes (under Review tab)
        i. One purpose: to ensure the transcript is not being altered
        ii. For the new coding method: to make your typed-in annotations distinct form the original transcript!
2. **Segment transcript**
    a. First, read all sections of the transcript
    b. For each section, segment into detail units
        i. Levine: "the scorer […] divides the entire response (including information external to the main event) into small segments (details). These segments are assigned various category labels. Finally, several qualitative ratings are assigned."
        ii. <u>Set the highlight tool to grey color</u>
        iii. Highlight what you consider to be the smallest meaningful unit possible
            1. Levine (2002): "A segment, or detail, is an information bit; it is a unique occurrence, observation, fact, statement, or thought. This will usually be a grammatical clause -- a sentence or part of a sentence that independently conveys information (i.e., a subject and a predicate), although a single clause may contain more than one detail. For each clause, consider whether its constituent parts convey additional information. If so, the parts can be separated and scored as separate segments. For example, the statement "he had an old, brown fedora" would receive two perceptual details because the term "old" significantly alters the meaning of the brown fedora, which on its own would receive one detail. If it is hard to do that (i.e. if multiple meaningful units are mixed in one section), highlight the larger group of units, but annotate that there are multiple details (next step…)"

See: Levine, B., Svoboda, E., Hay, J. F., Winocur, G., & Moscovitch, M. (2002). Aging and autobiographical memory: Dissociating episodic from semantic retrieval. *Psychology and Aging*, *17*(4), 677–689.

        iv. In line with the text, directly to the right of the grey-highlighted segment, type

the **number of details** in that highlighted segment (e.g. 1, 2), and/or:

1. If detail is redundant with previous highlights, this is a **Repetition** -- re-highlight in red
    a. From Levine: "Repetitions. A detail is a repetition if it is an unsolicited repetition of a prior information-containing detail. It does not have to be a verbatim repetition, but it should not add any new information to the prior detail ("I hoped for the best. I kept my fingers crossed" -- second sentence is a repetition). Score all repetitions, even if they are part of normal discourse […] Repetitions must convey information (as opposed to just words that are repeated). In the example below, "… and stuff" is repeated, but there is no information in this utterance, so it is not considered a repetition. As well, only score repetitions when they convey the same information as in an earlier detail. In the example below, "They really really liked me" is not a repetition of "They were happy with my work." Similarly, "I was a carpenter's helper", "I helped them", and "They could depend on me" are all different. "They liked what I did" however is the same as "They liked my work." Then he repeats this repetition straight away. Note that repetitions are scored across the three cueing conditions. In other words, details mentioned in free recall are scored as repetitions if they are repeated in specific probing (unless they were clearly prompted by the examiner's query).
2. If sentence and/or clause begins with "I think," "I guess," or "maybe," or anything else expressing some lack of confidence in what is being recalled, abbreviate **low confidence** as LC (e.g. "2, LC")

3. **Verifiable details (incl. low confidence and external details)**
    a. For each section, refer to the correct sideplot versions in the original narrative
        i. Go segment by segment
        ii. For each detail unit that can be directly verified within the sideplots, re-highlight the segment in green
            1. Mark **which story section** the detail(s) can be verified from (e.g. "1, 1B"). If can be verified *within either* section, mark both sideplots (e.g. "1, either 1B or 2A"). If detail is verifiable, but incorporates information from *both* sideplots, mark as Integrated (e.g. "1, integrated")
                a. If coding Charles or Karen, can apply this scheme to which

Charles or Karen segment is involved (e.g. 1A, either 1B or 2C, integrated, etc).

b. UPDATE 11/2017: Charles and Karen segments have new, finer grained segmentation (e.g. 1A, 2D, etc instead of 1 vs 2). If a detail is "Either" or "Integrated," be sure to indicate whether that pertains to multiple segments within a specific story, or across both stories for that main character (e.g. "Either 1," "Integrated 1+2").

2. If marked as "**Low confidence**" before, should re-highlight that segment in yellow instead
3. If Verifiable details pertain to a character that is not the character prompted (e.g. talking about events/details involving Charles when Melvin is the cue), but verifiable from other parts of the story, mark as **External Verifiable --** re-highlight in light blue/cyan
    a. Although this is not exactly "external," when coding for Charles or Karen, please mark details about interactions with other cued side-characters (e.g. Melvin) as External

b. **Important:** If participants simply restate the relation of the characters to other characters (as given in the cued recall prompt), count as "Other" -- re-highlight in magenta

4. **Everything else: Other (w/ comments)**
    a. Any remaining "details" which could not be coded are **Other** -- re-highlight in magenta
    b. If Other is a **Character Switch**, write it in (e.g. 2, Character Switch)
    c. If you notice anything else (maybe weird things), add in a New Comment bubble and type what you noticed
5. **Additional stipulations**
    a. You may have certain details within a segment which are verifiable, certain which aren't, etc. You can re-highlight an initially grey segment in multiple colors, and your annotations can be separated by a semicolon (e.g. if you have a segment highlighted in magenta and then green, can say something like, "1; 1, 2A").
    b. After all of the above -- there should be no grey highlights left. Please double-check.
6. **Data tabulation**
    a. **Open spreadsheet: "[Initials]_sideplots_annotated_[date]"**
    b. **For each segment, count the number of each detail type, type into appropriate cell**
        i. E.g. if counting details for Sub3 version 6, Beatrice:
            1. Verifiable details or Low confidence verifiable details could be specific to sideplot 1 or 2, drawn from Either, or Integrated (see

instructions for coding Verifiable details, earlier in this document)

2. All other detail categories would be placed on the Either line
3. If characters were confused (e.g. if information about Sandra is recalled for Beatrice), comment in the Character Switch column
4. Use Comments cell to describe anything else that came up

c. **Open spreadsheet: "[Initials]_mainplots_annotated_date'"**

   i. **Follow same instructions as for sideplots, but for main plot sections**
