# Supplementary material for: Narratives bridge the divide between distant events in episodic memory
Source: Mem Cognit. 2021 Apr 26;50(3):478–94. doi: 10.3758/s13421-021-01178-x (PMC8546012; doi:10.3758/s13421-021-01178-x)
Supplement: Supplementary file 4 — (PDF 86 kb) [file 13421_2021_1178_MOESM4_ESM.pdf]

Supplementary Data 3: Examples of one participant's recall with and without scoring.

Without Scoring:

Beatrice 1A/Beatrice 2A

shes a black woman who calls charles becuase of some issue and he gioves her advice I think it was about her fighting with her girlfriend because she didnt want a lab with a pink bow. In the next story, shes seen crying because her girlfriend ended up getting the dog and now she has to move out and is looking for a single room appartment

Melvin 1A/Melvin 2B

He is trying to audition ofr a role in shakespeares play hamlet. Charles sees him at the mayors speech thing and hes wearing a frilly neck collar. He says that hes auditioning to play hamlet or ophelia and gives a brief example. I think this may be right but I'm not sure but hes in the second story as well,

Sandra 1B/Sandra 2A

She is sitting at the table at the french restaurant when we first meet her. She was stood up on a date and then slaps the guy when he finally walks into the resturant because karen says she should have more respect for herself.

Johnny 1A/Johnny 2A

He was scrambling to find his mash potato recipe that his grandma had made because he needed to please his boyfriend and make mash potatoes for something special. Later, he calls Karen because he needs a flight to hawaii and wants to know if she knew a good one. Apparently, his boyfriend felt like he was too harsh on him and wanted to treat him with a trip

Charles

Charles Bort is wearing a purple scarf. He wants to get promoted to Head edititor of photography, and he knows he can do it if he gets this one picture of the crooked mayor. He gets a phone call from his cousin beatrice who is asking advice foir her and her girlfriend about getting a dog. He sugggests that they go to the pound together and pick one out. His boss tells him that the mayor is going to be speaking in 20 minutes and thsat he hasd to get there asap. He runs out and makes it intime and all the other reporters are already there and he strikes up a conversation with one of them. Then he sees his friend martin doyle wearing a 1800s collar and he tells him that hes auditioning for hamlet. The major arrives from dropping his daugter off at school and a reporter asks him a question about him being crooked and bfore he can answer, bort asks "smile mr. mayor!" and he snaps a photo aqs the major frowns. It the perfect photo to tie the whole corruption story together. Later, he is getting lunch with his mother, ethal. They sit snd split homemade sandwhiches on a picnic like thing. Hes wearing a barrett. He asks his mom if she saw his phot and she responds that she hasn't and he get really flustered and loads it on his super slow smart phone and is dismayed to see that no photo is there. he leaves and runs down to

talk to his boss but his boss is in a meeting, or so the receptionist tells him and he sits fuming when he sees that the evening paper is already printed. At some point he sees his coworker scarmbling to find his mash potatoe recipe for hos boyfriend. Hi9s boss comes out of the meeting and bort demands to know why his photo wasn't used. The boss who smokes a cigar and say "charlie boy!" says that he never got any photo and had to pay to get one from the other reporters. Charlie realizes that his phone never pressed sent and is dismayed that he won't get the promotion.

Karen

She was wearing silver earings. We first meet her when she is riding in a sweltering taxi cab on leather seats to go get lunch with her friend, Will (who is a black gentleman). Along the way she gets in to a discussion with the taxi driver on what he did for a living. She tells him that she just5 graduated from culinary school and is looking for a job with no luck. She gets a call from that other guys coworker who offwers some mash potatoes and asks for some inforamtion. When she gets top the lunch place, they order hot chocolate and cheese cake. Will says that he may have a job for her because hes friends with the manager at this french resturant where he had a reception once and they are hiring. Karen thanks him. Karen parks her car for two seconds to run some errands and when she comes back, it has been towed. She runs down to the tow place and after waiting in this really long annoying line, they finally open up a second station and she tells the attendant that her car is a black honda and can they please hurry up? The attendant says that the car blew up and so fill out this paperwork. She catches a bus to the restaurant and arrives late. After chatting with the manager, she sees her friend from yoga sitting at the resturant table oh and before that in the lunch place she sees her neighbor beatrice look really sad and beatrice tells her that she had to move out because her girlfriend goit a dog with a pink bow and if she knows any sdingle room apartemnts. But any way at the french place karen sees her friend alone and finds out that she was stood up on a date. The guy with long hair and leather jacket walks in and he gets slapped by yoga friend, sandra mayes. so then the manager says that renee is ready to meet with chef renne that is is ready to meet with her so she heads down to the kitchen and chef renee is so mad that she says how dare you walk in late to my kitchen! and Karen says that her car blew up but renee doens't care, so Karen flips her the finger and walks out and is it here that she sees sandra? I'm not sure. and I think thats it no happy ending

With Scoring: *Verifiable Details are highlighted in green, Low Confidence Verifiable Details are highlighted in yellow, External Verifiable Details are highlighted in Cyan (i.e. related to non-cued character events), Repetitions are highlighted in red, and Other details are highlighted in magenta. Semicolons separate different detail types that fall within an individual segment.*

Beatrice 1A/Beatrice 2A

shes a black woman 2 who calls charlesbecuase of some issue 2, 1A and he gioves her advice 1, 1A I think it was about her fighting with her girlfriend 2 LC, 1A because she didnt want a lab with a pink bow 2, 1A; 1. In the next story, shes seen crying 1 because her girlfriend ended up getting the dog 2, integrated 1A + 2A and now she has to move out 1, integrated 1A + 2A and is looking for a single room appartment 2, 2A

Melvin 1A/Melvin 2B

He is trying to audition 1, 1A ofr a role in shakespeares play hamlet. 3, 1A Charles sees him at the mayors speech thing 1, 1A and hes wearing a frilly neck collar. 2, 1A He says that hes auditioning to play hamlet or Ophelia 2, 1A and gives a brief example 1, 1A. I think this may be right but I'm not sure but hes in the second story as well, 1 LC

Sandra 1B/Sandra 2A

She is sitting at the table 1, 2A at the french restaurant 2, 2A when we first meet her 1. She was stood up 1 on a date 1, 2A and then slaps 1, 2A the guy 1, 2A when he finally walks into the resturant 2, 2A because karen says she should have more respect for herself. 1; 1, 2A

Johnny 1A/Johnny 2A

He was scrambling 1, 1A to find 1, 1A his mash potato recipe 1, 1A that his grandma had made 1, 1A because he needed to please his boyfriend 2, 1A and make mash potatoes 1, 1A for something special 1 Later, he calls Karen 1 because he needs a flight 1, 2A to hawaii 1, 2A and wants to know if she knew a good one. 1, 2A Apparently, his boyfriend felt like he was too harsh on him 2, 2A and wanted to treat him 1, 2A with a trip 1, 2A

Charles

Charles Bort is wearing a purple scarf. 2, 1A He wants to get promoted to Head edititor of photography 2, Integrated 2A + 2C; 1 and he knows he can do it 1, Integrated 1A + 1F + 2A + 2C if he gets this one picture 1, 1A of the crooked mayor. 1, 1D He gets a phone call 1 from his cousin beatrice 1 who is asking advice foir her and her girlfriend about getting a dog. 3 He sugggests that they go to the pound together and pick one out. 3 His boss tells him that the mayor is going to be speaking in 20 minutes 4, 1C and thsat he hasd to get there asap 2, 1C. He runs out and makes it intime 1; 1, Integrated 1C + 1D and all the other reporters are already there 1, 1D and he strikes up a conversation with one of them 2, 1D. Then he sees his friend martin doyle 1 wearing a 1800s collar 1 and he tells him that hes auditioning for hamlet 2The major arrives 1, 1F from dropping his daugter off at school 2, 1D and a reporter asks him a question 1, 1F about

him being crooked 2 and bfore he can answer, bort asks "smile mr. mayor!" 3, 1F and he snaps a photo 1, 1F aqs the major frowns. 1 It the perfect photo 1, 1F to tie the whole corruption story together 1, 2C, 1. Later, he is getting lunch 1, 2A with his mother, ethal 2, 2A. They sit 1, 2A snd split homemade sandwhiches 1, 2A; 1 on a picnic like thing 1. Hes wearing a barrett 1, 2A. He asks his mom 1, 2C if she saw his phot 1, 2C and she responds that she hasn't 1, 2C and he get really flustered 2 and loads it 1, 2C on his super slow smart phone 3, 2C and is dismayed to see that no photo is there 2, 2C. he leaves 1, 2D and runs down to talk to his boss 3, 2D but his boss is in a meeting 1, 2D or so the receptionist tells him 1, 2D and he sits fuming 1 when he sees that the evening paper is already printed 3, 2D. At some point he sees his coworker 1 scarmbling to find his mash potatoe recipe 2 for hos boyfriend 1. Hi9s boss comes out of the meeting 2, 2F and bort demands to know why his photo wasn't used 2, 2F. The boss who smokes a cigar 1;1, 2F;1 and say "charlie boy!" 1, 2F says that he never got any photo 2, 2F and had to pay to get one from the other reporters 2. Charlie realizes that his phone never pressed sent 2, 2F and is dismayed that he won't get the promotion. 2, 2F

Karen

She was wearing silver earings. 2, 1A We first meet her when she is riding 2, 1A in a sweltering taxi cab 2, 1A on leather seats 1, 1A to go get lunch 1 with her friend,1, 1A Will 1, 1A (who is a black gentleman) 1. Along the way1, either 1A or 1C she gets in to a discussion1, either 1A or 1C with the taxi driver1, either 1A or 1C on what he did for a living1, 1C. She tells him that she just5 graduated1 from culinary school1A and is looking for a job1C with no luck1C. She gets a call1 from that other guys coworker1 who offwers some mash potatoes1 and asks for some inforamtion1. When she gets top the lunch place1, they order hot chocolate1, 1F and cheese cake.1,1F Will says that he may have a job for her1,1F because hes friends with the manager1,1F at this french resturant2,1F where he had a reception once1,1F and they are hiring.1,1F Karen thanks him. 1 Karen parks her car 1,2A for two seconds1 to run some errands1,2A and when she comes back,1,2A it has been towed.1,2A She runs down to the tow place1,2A and after waiting in this really long annoying line,2, integrated 2A+2C they finally open up a second station1, 2C and she tells the attendant1, 2C that her car is a black honda2, 2C and can they please hurry up? 1, 2C The attendant says that the car blew up2, 2C and so fill out this paperwork.1, 2C She catches a bus1, 2D to the restaurant1, 2D and arrives late.1, 2D After chatting with the manager,1, 2D she sees her friend from yoga1 sitting at the resturant table1 oh and before that in the lunch place1 she sees her neighbor beatrice look really sad1 and beatrice tells her that she had to move out1 because her girlfriend1 goit a dog with a pink bow2 and if she knows any sdingle room apartemnts.2 But any way at the french place 1 karen sees her friend alone 1 and finds out that she was stood up on a date.2 The guy with long hair and leather jacket2 walks in1 and he gets slapped by yoga friend, sandra mayes.1 so then the manager says that renee is ready to meet with chef renne that is is ready to meet with her1, 2F so she heads down to the kitchen1,2F and chef renee is so mad1, 2F that she says how dare you walk in late to my kitchen! 3, 2F and Karen says that her car blew up1, 2F but renee doens't care1, 2F so Karen flips her the finger1, 2F and walks out1, 2F and is it here that she sees sandra? I'm not sure.1 and I think thats it no happy ending1
